# PGE_2_ limits effector expansion of tumour-infiltrating stem-like CD8^+^ T cells

**DOI:** 10.1038/s41586-024-07254-x

**Published:** 2024-04-24

**Authors:** Sebastian B. Lacher, Janina Dörr, Gustavo P. de Almeida, Julian Hönninger, Felix Bayerl, Anna Hirschberger, Anna-Marie Pedde, Philippa Meiser, Lukas Ramsauer, Thomas J. Rudolph, Nadine Spranger, Matteo Morotti, Alizee J. Grimm, Sebastian Jarosch, Arman Oner, Lisa Gregor, Stefanie Lesch, Stefanos Michaelides, Luisa Fertig, Daria Briukhovetska, Lina Majed, Sophia Stock, Dirk H. Busch, Veit R. Buchholz, Percy A. Knolle, Dietmar Zehn, Denarda Dangaj Laniti, Sebastian Kobold, Jan P. Böttcher

**Affiliations:** 1 Institute of Molecular Immunology, School of Medicine and Health, Technical University of Munich (TUM), Munich, Germany; 2 Division of Clinical Pharmacology, Department of Medicine IV, LMU University Hospital, Member of the German Center for Lung Research (DZL), LMU Munich, Munich, Germany; 3 Division of Animal Physiology and Immunology, School of Life Sciences Weihenstephan, TUM, Freising, Germany; 4 Institute for Medical Microbiology, Immunology and Hygiene, School of Medicine and Health, TUM, Munich, Germany; 5 Ludwig Institute for Cancer Research, Lausanne Branch, University of Lausanne (UNIL), Lausanne, Switzerland; 6 Department of Oncology, University Hospital of Lausanne (CHUV) and UNIL, Lausanne, Switzerland; 7 Agora Cancer Research Center, Lausanne, Switzerland; 8 Department of Medicine III, LMU University Hospital, LMU Munich, Munich, Germany; 9 German Cancer Consortium (DKTK), partner site Munich, a partnership between DKFZ and LMU University Hospital, Munich, Germany; 10 Einheit für Klinische Pharmakologie (EKLiP), Helmholtz Munich, Research Center for Environmental Health (HMGU), Neuherberg, Germany; 11 Present Address: Boehringer Ingelheim, Biberach, Germany

**Keywords:** Tumour immunology, Cancer microenvironment, Immunotherapy

## Abstract

Cancer-specific TCF1^+^ stem-like CD8^+^ T cells can drive protective anticancer immunity through expansion and effector cell differentiation^1–4^; however, this response is dysfunctional in tumours. Current cancer immunotherapies^2,5–9^ can promote anticancer responses through TCF1^+^ stem-like CD8^+^ T cells in some but not all patients. This variation points towards currently ill-defined mechanisms that limit TCF1^+^CD8^+^ T cell-mediated anticancer immunity. Here we demonstrate that tumour-derived prostaglandin E2 (PGE_2_) restricts the proliferative expansion and effector differentiation of TCF1^+^CD8^+^ T cells within tumours, which promotes cancer immune escape. PGE_2_ does not affect the priming of TCF1^+^CD8^+^ T cells in draining lymph nodes. PGE_2_ acts through EP_2_ and EP_4_ (EP_2_/EP_4_) receptor signalling in CD8^+^ T cells to limit the intratumoural generation of early and late effector T cell populations that originate from TCF1^+^ tumour-infiltrating CD8^+^ T lymphocytes (TILs). Ablation of EP_2_/EP_4_ signalling in cancer-specific CD8^+^ T cells rescues their expansion and effector differentiation within tumours and leads to tumour elimination in multiple mouse cancer models. Mechanistically, suppression of the interleukin-2 (IL-2) signalling pathway underlies the PGE_2_-mediated inhibition of TCF1^+^ TIL responses. Altogether, we uncover a key mechanism that restricts the IL-2 responsiveness of TCF1^+^ TILs and prevents anticancer T cell responses that originate from these cells. This study identifies the PGE_2_–EP_2_/EP_4_ axis as a molecular target to restore IL-2 responsiveness in anticancer TILs to achieve cancer immune control.

## Abstract (web summary)

Tumour-derived prostaglandin E_2_, signaling through its receptors EP_2_ and EP_4_, is shown to restrain the responses of tumour-infiltrating stem-like TCF1^+^CD8^+^ T lymphocytes, and modulation of T cell EP_2_ and EP_4_ can restore anticancer immunity.

## Main

Increased production of the bioactive lipid PGE_2_ downstream of aberrant cyclooxygenase 1 (COX1; encoded by *Ptgs1*) and COX2 (encoded by *Ptgs2*) activity is observed in many human tumours and is associated with cancer progression and poor patient survival^10–13^. Studies using preclinical mouse cancer models have demonstrated that tumour-derived PGE_2_ has an important role in tumour escape from anticancer immunity^14,15^. PGE_2_ signalling is mediated by four G protein-coupled receptors that are broadly expressed on various immune cell populations, EP_1_, EP_2_, EP_3_ and EP_4_ (encoded by *PTGER1*, *PTGER2*, *PTGER3* and *PTGER4*, respectively), of which signalling through EP_2_/EP_4_ can suppress immune cell function^16^. Previous studies have implicated a role for PGE_2_ in the regulation of T cell biology and function^17–20^; however, the impact of PGE_2_ on TCF1^+^CD8^+^ T cells and their ability to mount protective anticancer responses remains unclear.

## The PGE_2_–EP_2_/EP_4_ axis controls anticancer CD8^+^ T cell responses

**Fig. 1:**
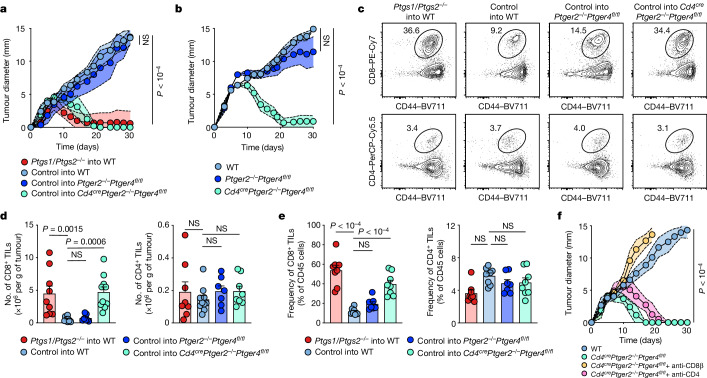
EP_2_/EP_4_ deficiency permits CD8^+^ T cell-mediated tumour immune control. **a**, Tumour growth profiles of 2 × 10^5^ *Ptgs1/Ptgs2*^*−/−*^ or control BRAF^V600E^ melanoma cells transplanted into WT mice, *Ptger2*^*−/−*^*Ptger4*^*fl/fl*^ mice and *Cd4*^*cre*^*Ptger2*^*−/−*^*Ptger4*^*fl/fl*^ mice (*n* = 10 each). **b**, Growth profiles of 2 × 10^6^ Panc02 cells transplanted into WT mice, *Ptger2*^*−/−*^*Ptger4*^*fl/fl*^ mice and *Cd4*^*cre*^*Ptger2*^*−/−*^*Ptger4*^*fl/fl*^ mice (*n* = 8 each). **c**–**e**, WT mice, *Ptger2*^*−/−*^*Ptger4*^*fl/fl*^ mice and *Cd4*^*cre*^*Ptger2*^*−/−*^*Ptger4*^*fl/fl*^ mice were subcutaneously (s.c.) injected with 2 × 10^6^ control or *Ptgs1/Ptgs2*^*−/−*^ BRAF^V600E^ cells and TILs were analysed 11 days later by flow cytometry. **c**, Plots showing the frequencies of CD8^+^ and CD4^+^ TILs among CD45^+^ immune cells and expression of the activation marker CD44. **d**, Quantification of TIL numbers (CD8^+^ TILs: *Ptgs1/Ptgs2*^*−/−*^ into WT, *n* = 9; control into WT, *n* = 10; control into *Ptger2*^*−/−*^*Ptger4*^*fl/fl*^, *n* = 7; control into *Cd4*^*cre*^*Ptger2*^*−/−*^*Ptger4*^*fl/fl*^, *n* = 10; CD4^+^ TILs: *Ptgs1/Ptgs2*^*−/−*^ into WT, *n* = 8; control into WT, *n* = 10; control into *Ptger2*^*−/−*^*Ptger4*^*fl/fl*^; *n* = 7; control into *Cd4*^*cre*^*Ptger2*^*−/−*^*Ptger4*^*fl/fl*^, *n* = 8). **e**, TIL frequencies (*Ptgs1/Ptgs2*^*−/−*^ into WT, *n* = 8; control into WT, *n* = 8; control into *Ptger2*^*−/−*^*Ptger4*^*fl/fl*^, *n* = 7; control into *Cd4*^*cre*^*Ptger2*^*−/−*^*Ptger4*^*fl/fl*^, *n* = 8). **f**, Effect of CD8^+^ and CD4^+^ T cell depletion on control BRAF^V600E^ tumour growth in *Cd4*^*cre*^*Ptger2*^*−/−*^*Ptger4*^*fl/fl*^ mice (*Cd4*^*cre*^*Ptger2*^*−/−*^*Ptger4*^*fl/fl*^, *n* = 8; WT, *n* = 9). Data in **a**, **b** and **d**–**f** are pooled from two (**b**,**f**) or three (**a**,**d**,**e**) independent experiments and depicted as the mean ± s.e.m. Plots in **c** show data for 1 tumour representative of *n* = 7 tumours from 2 independent experiments. *P* values are from two-way analysis of variance (ANOVA) with Bonferroni’s correction for multiple testing (**a**,**b**,**f**) or one-way ANOVA with Dunnett’s multiple-comparison test (**d**,**e**). NS, not significant (*P* ≥ 0.05). Source Data

**Extended Data Fig. 1:**
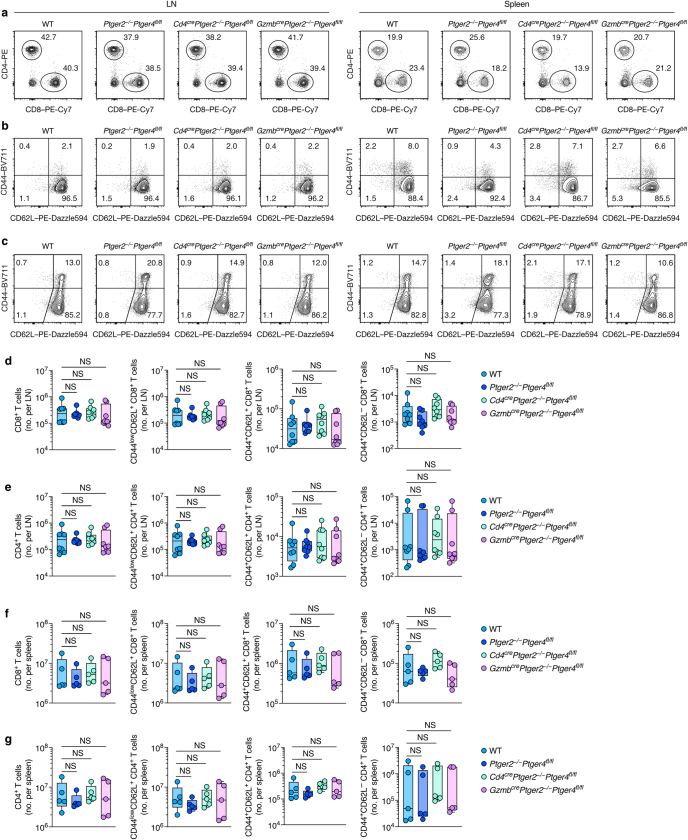
*Cd4*^*cre*^*Ptger2*^*−/−*^*Ptger4*^*fl/fl*^, *Gzmb*^*cre*^*Ptger2*^*−/−*^*Ptger4*^*fl/fl*^ and *Ptger2*^*−/−*^*Ptger4*^*fl*/*fl*^ mice exhibit normal T cell profiles. Flow cytometric analysis of CD8^+^ and CD4^+^ T cells in spleens and inguinal lymph nodes (LN) of WT, *Ptger2*^*−/−*^*Ptger4*^*fl/fl*^, *Cd4*^*cre*^*Ptger2*^*−/−*^*Ptger4*^*fl/fl*^ and *Gzmb*^*c*^*Ptger2*^*−/−*^*Ptger4*^*fl/fl*^ mice (n = 8 per group). **a**-**c**, Representative plots showing **a**, CD4^+^ and CD8^+^ T cell frequency among live CD45^+^ cells or T cell subset composition among **b**, CD4^+^ T cells or **c**, CD8^+^ T cells as measured by CD44 and CD62L marker expression. **d**-**g**, Quantification of total cell numbers and subset composition for **d**, CD8^+^ T cells in LN (n = 8 per group), **e**, CD4^+^ T cells in LN (n = 8 per group), **f**, splenic CD8^+^ T cells (n = 5 per group) and **g**, splenic CD4^+^ T cells (n = 5 per group), based on **a**-**c**. Plots in **a**-**c** show data for one sample representative for n = 5–8 samples from two independent experiments. Data in **d**-**g** are pooled from two independent experiments and depicted as box plots extending from the 25^th^ to 75^th^ percentiles with the median as centre and whiskers corresponding to minimum and maximum values. *P* ≥ 0.05, not significant (NS) as determined by one-way ANOVA with Dunnett’s multiple-comparison test. Source Data

**Extended Data Fig. 2:**
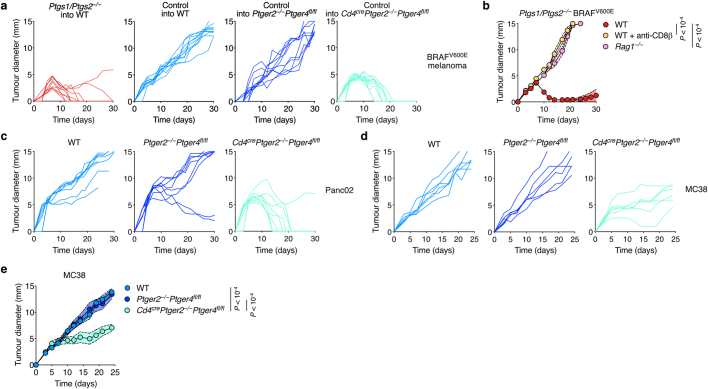
*Cd4*^*cre*^*Ptger2*^*−/−*^*Ptger4*^*fl*/*fl*^ mice reject PGE_2_-producing tumours. Growth profiles following s.c. inoculation of cancer cell lines into WT, *Ptger2*^*−/−*^*Ptger4*^*fl/fl*^ or *Cd4*^*cre*^*Ptger2*^*−/−*^*Ptger4*^*fl/fl*^ mice. **a**, Individual profiles of 2 × 10^5^ control or *Ptgs1/Ptgs2*^*−/−*^ BRAF^V600E^ tumour cells (n = 10). **b**, Growth of 2 × 10^5^ *Ptgs1/Ptgs2*^*−/−*^ BRAF^V600E^ melanoma cells transplanted into *Rag1*^*−/−*^ mice (n = 4) or WT mice with or without CD8^+^ T cell depletion (n = 5 per group). **c**, Individual profiles of 2 × 10^6^ Panc02 tumour cells (n = 8). **d**, Individual profiles of 2 × 10^5^ MC38 tumour cells (n = 6). **e**, Representation of all profiles shown in **d**. Data in **a**-**e** are pooled from two (**b**,**c**,**d**,**e**) or three (**a**) independent experiments and depicted as mean ± s.e.m. *P *values are from two-way ANOVA with Bonferroni’s multiple-comparison test. *P* ≥ 0.05, not significant (NS). Source Data

To determine the role of PGE_2_ in tumour escape from anticancer T cell responses, we generated *Cd4*^*cre*^*Ptger2*^*−/−*^*Ptger4*^*fl/fl*^ mice in which Cre recombinase activity induces the deletion of EP_4_ in CD4^+^ and CD8^+^ T cells on a global EP_2_-deficient background. We also generated additional control mice that lack only EP_2_ (*Ptger2*^*−/−*^*Ptger4*^*fl/fl*^ mice), which enabled the testing of possible effects of global EP_2_ deficiency. T cell profiling in *Cd4*^*cre*^*Ptger2*^*−/−*^*Ptger4*^*fl/fl*^ mice and *Ptger2*^*−/−*^*Ptger4*^*fl/fl*^ mice compared with C57BL/6 wild-type (WT) mice revealed normal CD4^+^ and CD8^+^ T cell abundance and subset composition in lymphoid organs (Extended Data Fig. 1a–g). Unaltered T cell composition was similarly observed in *Gzmb*^*cre*^*Ptger2*^*−/−*^*Ptger4*^*fl/fl*^ mice, which lack EP_2_ and EP_4_ in CD8^+^ T cells expressing granzyme B (GZMB) (Extended Data Fig. 1a–g). Notably, after tumour challenge, *Cd4*^*cre*^*Ptger2*^*−/−*^*Ptger4*^*fl/fl*^ mice exhibited improved tumour immune control, and fully rejected tumours formed by immune-evasive, PGE_2_-producing (control) BRAF^V600E^ melanoma cells (Fig. 1a and Extended Data Fig. 2a). This was not the case for *Ptger2*^*−/−*^*Ptger4*^*fl/fl*^ mice and WT mice, in which control BRAF^V600E^ tumours progressively grew (Fig. 1a and Extended Data Fig. 2a). We further validated that BRAF^V600E^ melanoma depended on tumour-derived PGE_2_ to evade anticancer immunity by demonstrating that COX-deficient *Ptgs1/Ptgs2*^*−/−*^ BRAF^V600E^ melanoma, which lacks PGE_2_ production, failed to escape immune control (Fig. 1a and Extended Data Fig. 2a). We also confirmed that this effect is mediated by CD8^+^ T cells^14^ (Extended Data Fig. 2b). Extending our analysis to other mouse tumour models, tumours formed by Panc02 pancreatic cancer cells similarly exhibited complete regression in *Cd4*^*cre*^*Ptger2*^*−/−*^*Ptger4*^*fl/fl*^ mice but not in *Ptger2*^*−/−*^*Ptger4*^*fl/fl*^ or WT mice (Fig. 1b and Extended Data Fig. 2c). Similar results were observed for tumours derived from MC38 colorectal cancer cells (Extended Data Fig. 2d,e).

Immune control of control BRAF^V600E^ melanoma tumours in *Cd4*^*cre*^*Ptger2*^*−/−*^*Ptger4*^*fl/fl*^ mice was linked to markedly increased CD8^+^ TIL accumulation (Fig. 1c–e). By contrast, no substantial differences were observed for CD4^+^ TILs (Fig. 1c–e). This result suggests that although PGE_2_–EP_2_/EP_4_ signalling may affect CD4^+^ T cell function, these cells, at least in BRAF^V600E^ tumours, do not have a major role in immune escape. Consistently, antibody-mediated T cell depletion confirmed the relevance of CD8^+^ but not CD4^+^ T cells for immune control of PGE_2_-producing BRAF^V600E^ tumours in *Cd4*^*cre*^*Ptger2*^*−/−*^*Ptger4*^*fl/fl*^ mice (Fig. 1f). Taken together, these data suggest that EP_2_/EP_4_ signalling controls the accumulation of CD8^+^ TILs in PGE_2_-producing tumours and that this is important for cancer immune evasion.

## PGE_2_ does not affect CD8^+^ T cell priming

**Extended Data Fig. 3:**
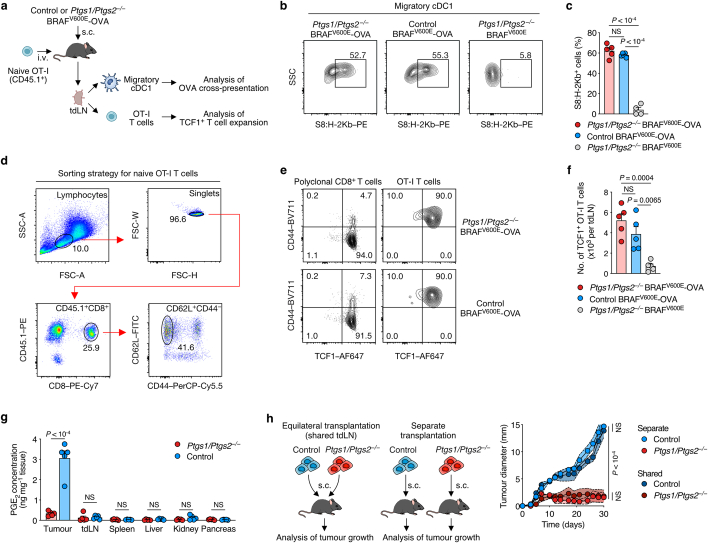
Tumour-derived PGE_2_ does not impact on priming of anticancer CD8^+^ T cells in lymph nodes. **a**, Experimental design for **b**-**e**. WT mice received 1 × 10^3^ naive OVA-specific CD45.1^+^ OT-I T cells followed by inoculation with 2 × 10^6^ control or *Ptgs1/Ptgs2*^*−/−*^ BRAF^V600E^-OVA cells. 6 days later, tdLNs were analysed by flow cytometry. Mice injected with *Ptgs1/Ptgs2*^*−/−*^ BRAF^V600E^ cells (lacking OVA expression) served as control. **b**, Representative plot showing S8:H-2K^b^ surface staining on migratory cDC1 (identified as live CD45^+^CD11c^+^MHCII^hi^CD103^+^CD8α^−^CD11b^−^ cells). **c**, Quantification based on **b**, with n = 5 for control and *Ptgs1/Ptgs2*^*−/−*^ BRAF^V600E^-OVA, n = 4 for *Ptgs1/Ptgs2*^*−/−*^BRAF^V600E^. **d**, Flow cytometry plots showing the sorting strategy for naive OT-I T cells. **e**, Flow cytometry plots showing expression of CD44 and TCF1 in polyclonal CD8^+^ T cells and OVA-specific OT-I T cells. **f**, Quantification of antigen-experienced CD44^+^ TCF1^+^ OT-I T cells based on **e**. n = 5. **g**, PGE_2_ concentration in lysates from tumours and indicated organs analysed 11 days after s.c. inoculation of WT mice with control or *Ptgs1/Ptgs2*^*−/−*^ BRAF^V600E^ melanoma cells (n = 5 per group). **h**, Effect of equilateral co-transplantation of 2 × 10^5^ control and *Ptgs1/Ptgs2*^*−/−*^ BRAF^V600E^ tumours on tumour growth (n = 4 per group). Data in **c** and **f**-**h** are pooled from two (**c**,**f**,**h**) or three (**g**) independent experiments and depicted as mean ± s.e.m. Plots in **b**,**e** show data for one sample representative for n = 5 samples from two independent experiments. *P *values in **c**,**f** are from one-way ANOVA with Tukey’s multiple-comparison test, *P *values in **g** are from unpaired t-tests, *P *values in **h** are from two-way ANOVA with Bonferroni’s multiple-comparison test. *P* ≥ 0.05, not significant (NS). Source Data

Priming of anticancer CD8^+^ T cells in tumour-draining lymph nodes (tdLNs) by type 1 conventional dendritic cells (cDC1s) that transport tumour antigens to tdLNs is thought to underlie anticancer CD8^+^ T cell responses^21,22^. To test whether PGE_2_ impairs cDC1-mediated CD8^+^ T cell priming, we injected WT mice with PGE_2_-producing control or PGE_2_-deficient *Ptgs1/Ptgs2*^*−/−*^ BRAF^V600E^ melanoma cells engineered to express the model antigen ovalbumin (OVA). We then determined the presence of migratory CD103^+^ cDC1 cross-presenting OVA-derived peptides on major histocompatibility complex (MHC) class I molecules in tdLNs (Extended Data Fig. 3a). Migratory cDC1s in both models cross-presented tumour-derived OVA protein with similar efficiency, as determined by staining for OVA(257–264) (SIINFEKL) peptide loading of the MHC class I molecule H-2K^b^ (Extended Data Fig. 3b,c). To further examine T cell priming, we adoptively transferred naive CD8^+^ OT-I T cells (Extended Data Fig. 3d), which express a transgenic T cell receptor (TCR) specific for OVA, into mice subsequently transplanted with BRAF^V600E^-OVA tumours. Naive (CD44^low^) OT-I T cells efficiently expanded into CD44^+^TCF1^+^ OT-I T cells within tdLNs in both groups (Extended Data Fig. 3e,f). This result demonstrates that T cell priming is unaffected. Consistent with these data, we did not detect substantial PGE_2_ levels in tdLNs from control BRAF^V600E^ tumours or in other distant organs (Extended Data Fig. 3g). Moreover, progressive outgrowth of control BRAF^V600E^ tumours and efficient immune control of *Ptgs1/Ptgs2*^*−/−*^ BRAF^V600E^ tumours was unchanged following tumour transplantation to the same lymph drainage site (Extended Data Fig. 3h). These findings imply that an anticancer CD8^+^ T cell response initiated in the shared tdLN achieves effective elimination of the PGE_2_-deficient tumour but nevertheless fails in the co-transplanted PGE_2_-producing tumour. Taken together, these data demonstrate that PGE_2_ controls anticancer CD8^+^ T cell responses locally within tumour tissue, which raises the question of how it affects CD8^+^ TILs.

## PGE_2_ controls CD8^+^ TIL effector expansion

CD8^+^ TILs are heterogenous and comprise at least two phenotypically and functionally distinct populations: (1) proliferation and differentiation competent TCF1^+^ cells that lack cytotoxic effector functions (often referred to as ‘stem-like’ or ‘precursor of exhausted’ T cells); and (2) TIM-3^+^(TCF1^−^) cells that encompass more differentiated effector and terminally differentiated or exhausted T cells. TCF1^+^CD8^+^ T cells fulfil an essential role in anticancer immunity by giving rise to TIM-3^+^ progeny through proliferative expansion and effector differentiation^2,5,8,9^. This process is pivotal for anticancer immunity that at least in part occurs locally within tumour tissue^1–3^.

**Fig. 2:**
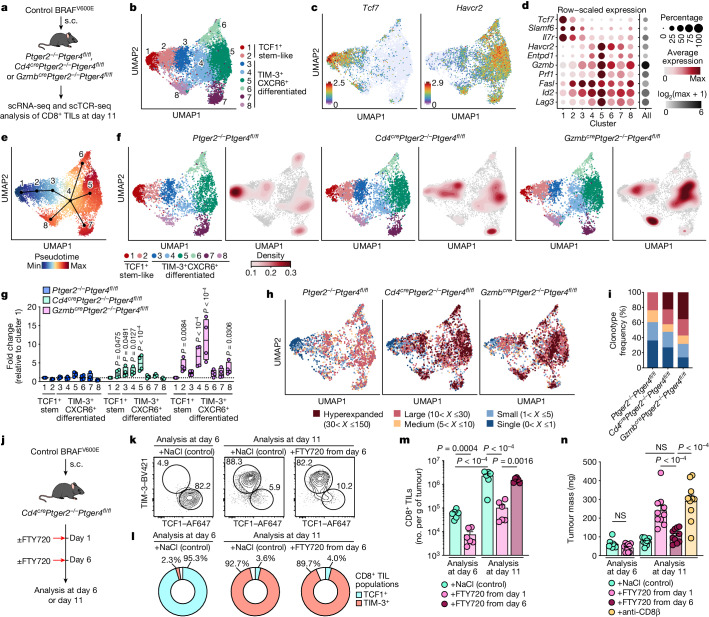
Ablation of T cell-intrinsic EP_2_/EP_4_ signalling rescues CD8^+^ T cell expansion and effector differentiation in PGE_2_-producing tumours. **a**–**g**, scRNA-seq analyses of CD8^+^ TILs in control BRAF^V600E^ tumours from *Ptger2*^*−/−*^*Ptger4*^*fl/fl*^ mice, *Cd4*^*cre*^*Ptger2*^*−/−*^*Ptger4*^*fl/fl*^ mice and *Gzmb*^*ce*^*Ptger2*^*−/−*^*Ptger4*^*fl/fl*^ mice (*n* = 4 each). **a**, Experimental design. **b**, Uniform manifold approximation and projection (UMAP) plot of 12,516 CD8^+^ TILs coloured according to cluster classification. **c**, Visualization of *Tcf7* and *Havcr2* transcript levels. **d**, PCPT plot showing expression levels of selected genes. **e**, Developmental trajectory prediction by unsupervised slingshot analysis. **f**,**g**, Comparison of CD8^+^ TIL clusters among *Ptger2*^*−/−*^*Ptger4*^*fl/fl*^ mice, *Cd4*^*cre*^*Ptger2*^*−/−*^*Ptger4*^*fl/fl*^ mice and *Gzmb*^*cre*^*Ptger2*^*−/−*^*Ptger4*^*fl/fl*^ mice. **f**, Density analysis. **g**, Quantification relative to cluster 1 (*n* = 4 each). **h**,**i**, scTCR-seq analyses of CD8^+^ TILs from *n* = 3 tumours for each group. **h**, UMAP visualizations of T cell clonotype distribution. **i**, Quantification of T cell clonotype frequency. **j**–**n**, TIM-3^+^ effector CD8^+^ T cell differentiation in tumour tissue. *Cd4*^*cre*^*Ptger2*^*−/−*^*Ptger4*^*fl/fl*^ mice bearing control BRAF^V600E^ tumours were injected with FTY720 or NaCl as control. **j**, Experimental design. **k**, Representative flow cytometry plots showing TCF1 and TIM-3 expression among CD44^+^CD8^+^ TILs. **l**, Average percentages of CD8^+^ TIL populations across *n* = 6 tumours. **m**, Quantification of CD8^+^ TIL numbers (*n* = 6). **n**, Analysis of tumour mass (*n* = 10). Anti-CD8β, antibody-mediated CD8^+^ T cell depletion in the absence of FTY720 treatment. Data in **a**–**h** are from one experiment. Data in **g** are depicted as box plots extending from the 25th to 75th percentiles with the median as the centre and the whiskers corresponding to the minimum and maximum values. Data in **k–n** are pooled from two (**k**,**l**,**m**) or three (**n**) independent experiments and depicted as the mean ± s.e.m. *P* values are from two-way ANOVA with Bonferroni’s correction for multiple testing (**g**) or one-way ANOVA with Tukey’s multiple-comparison test (**m**,**n**). Plots in **k** show data for 1 tumour representative for *n* = 6 tumours from 2 independent experiments. Source Data

**Extended Data Fig. 4:**
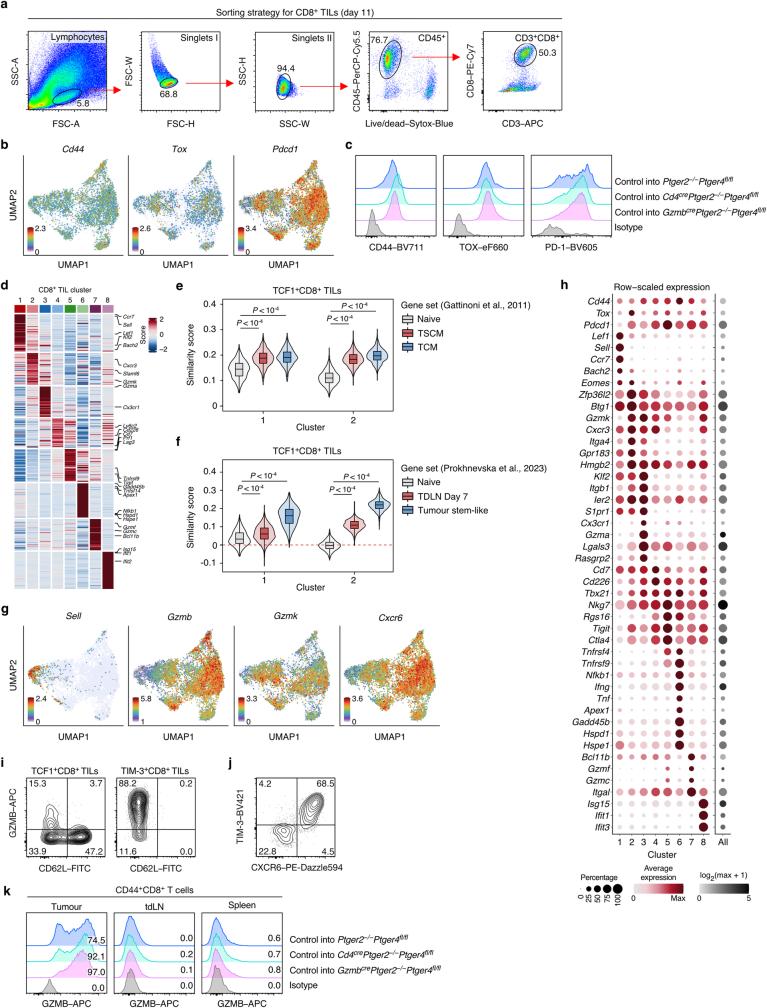
Isolation of CD8^+^ TILs for scRNA-seq and phenotypic characterisation of CD8^+^ TIL populations. *Ptger2*^*−/−*^*Ptger4*^*fl/fl*^, *Cd4*^*cre*^*Ptger2*^*−/−*^*Ptger4*^*fl/fl*^ or *Gzmb*^*cre*^*Ptger2*^*−/−*^*Ptger4*^*fl/fl*^ mice were transplanted with 2 × 10^6^ control BRAF^V600E^ melanoma cells. After 11 days, CD8^+^ TIL populations were sorted from n = 4 tumours per group and analysed by scRNA-seq (**b**,**d**-**h**) or flow cytometry (**c,i**-**k**). **a**, Flow cytometry plots showing the sorting strategy. **b**, UMAP plots showing transcript expression of *Cd44*, *Tox* and *Pdcd1* (encoding PD-1) as determined by scRNA-seq. **c**, Flow cytometric analysis of CD44, TOX and PD-1 protein expression in CD8^+^ TILs. **d**, Heatmap showing the expression of cluster signature transcripts (top 50). **e**,**f**, Correlation of TCF1^+^CD8^+^ TIL cluster gene expression with gene signatures of **e** naive CD8^+^ T cells, memory stem cell CD8^+^ T cells (TSCM) and central memory CD8^+^ T cells (TCM) or **f** naive CD8^+^ T cells, tumour antigen-specific CD8^+^ T cells in tdLNs and tumour-infiltrating stem-like CD8^+^ T cells. **g**, UMAP visualisation of transcript expression of indicated immune genes as determined by scRNA-seq. **h**, Expression levels of selected immune genes across CD8^+^ TIL clusters. **i**, Flow cytometric analysis of GZMB and CD62L expression among TCF1^+^ and TIM-3^+^ CD8^+^ TIL populations from a WT mouse. **j**, Flow cytometric analysis of TIM-3 and CXCR6 protein expression in CD8^+^ TILs from a WT mouse. **k**, Analysis of GZMB expression in activated (CD44^+^) CD8^+^ T cells isolated from tumours, tdLNs and spleen. Numbers indicate percentage of GZMB^+^ cells compared to isotype control. Plots in **a**,**c**,**i**-**k** show data for one tumour representative for n = 6 tumours from one (**a**,**b**), two (**c**,**i**,**k**), or three (**j**) independent experiments or pooled data from n = 4 biological replicates from one experiment (**d**-**h**). *P *values in **e**,**f** are from pairwise comparisons using Wilcoxon rank sum test and Bonferroni correction for multiple testing. *P* ≥ 0.05, not significant (NS).

Our results raised the question of whether interference with effector differentiation of TCF1^+^CD8^+^ TILs underlies the PGE_2_-mediated impairment of anticancer immunity. To address this issue across the single-cell landscape of CD8^+^ TILs, we performed parallel single-cell RNA sequencing (scRNA-seq) and single-cell TCR sequencing (scTCR-seq) of CD8^+^ TILs sorted from BRAF^V600E^ tumours at day 11 after tumour transplantation into *Cd4*^*cre*^*Ptger2*^*−/−*^*Ptger4*^*fl/fl*^ mice and *Ptger2*^*−/−*^*Ptger4*^*fl/fl*^ mice (as control) (Fig. 2a and Extended Data Fig. 4a). We also included *Gzmb*^*cre*^*Ptger2*^*−/−*^*Ptger4*^*fl/fl*^ mice (Fig. 2a), reasoning that this would enable us to determine the impact of EP_2_/EP_4_-mediated PGE_2_ signalling on those CD8^+^ T cells undergoing effector differentiation within tumour tissue. We further included four biological replicates in each group to ensure that heterogeneity among individual tumours is reflected in our analysis. scRNA-seq analysis revealed eight TIL clusters (Fig. 2b) that all expressed *Pdcd1* (which encodes PD-1), the activation marker *Cd44* and the transcription factor (TF) *Tox* (Extended Data Fig. 4b), a result consistent with their activation history. Of note, CD8^+^ TILs displayed equally high protein expression of CD44, TOX and PD-1 that did not differ among cells isolated from tumours in *Ptger2*^*−/−*^*Ptger4*^*fl/fl*^ mice, *Cd4*^*cre*^*Ptger2*^*−/−*^*Ptger4*^*fl/fl*^ mice and *Gzmb*^*cre*^*Ptger2*^*−/−*^*Ptger4*^*fl/fl*^ mice (Extended Data Fig. 4c).

In our concatenated scRNA-seq data, TIL clusters 1 and 2 shared high expression of stem-like T cell markers such as *Tcf7* (which encodes TCF1), *Slamf6* and *Il7r* (Fig. 2c,d and Extended Data Fig. 4d). Of note, both of these clusters displayed markedly higher expression of gene signatures of memory or tumour-reactive T cells than signatures for naive T cells (Extended Data Fig. 4e,f), which indicated that at least a substantial fraction of these cells is antigen-experienced. TCF1^+^ TILs in cluster 1 displayed enriched expression of *Sell* (which encodes CD62L), *Ccr7* and *Bach2* (Extended Data Fig. 4d,g,h). By contrast, TCF1^+^ TILs in cluster 2 lacked *Sell* but showed expression of markers associated with effector function (such as *Gzmb*, *Gzmk* and *Fasl*) and migration (*S1pr1*, *Itga4*, *Gpr183*, *Itgb1*, *Cxcr3* and *Ier2*) (Fig. 2d and Extended Data Fig. 4d,g,h), which indicated their incipient effector differentiation. Consistently, CD62L^−^TCF1^+^ TILs but not CD62L^+^TCF1^+^ TILs stained positive for intracellular GZMB protein (Extended Data Fig. 4i), although GZMB expression in these cells was low both in terms of frequency of GZMB^+^ cells and total GZMB levels. The remaining scRNA-seq clusters (clusters 3–8) lacked *Tcf7* expression and, in addition to *Gzmb*, shared expression of genes associated with T cell differentiation and effector function, including *Havcr2* (which encodes TIM-3) and high expression of the chemokine receptor *Cxcr6* (Fig. 2c,d and Extended Data Fig. 4g,h), which identified them as more differentiated early and/or terminally differentiated TIL populations. We confirmed co-expression of TIM-3 and CXCR6 at the protein level (Extended Data Fig. 4j) and used both molecules as overarching markers to collectively denote (TCF1^*−*^) effector TILs. Among the different clusters of TIM-3^+^CXCR6^+^ TILs, clusters 3 and 4 were marked by high expression of molecules associated with early effector-like cells; for example, *Cx3cr1* (refs. ^23,24^) in cluster 3 and *Cd7* (ref. ^25^) in cluster 4 (Extended Data Fig. 4d,h). By contrast, clusters 5–8 displayed increased expression of cytotoxic effector molecules and immune-inhibitory receptors (Fig. 2d and Extended Data Fig. 4h), but were distinguished by differential expression of cytokines (for example, *Ifng* and *Tnf*), molecules associated with growth arrest and DNA repair (*Apex1* and *Gadd45b*) and type I interferon signalling (*Isg15*, *Ifit1* and *Ifit3*) (Extended Data Fig. 4d,h). Notably, in contrast to tumour tissue, we did not detect any GZMB^+^ cells among activated CD44^+^CD8^+^ T cells in tdLNs or spleen of tumour-bearing mice (Extended Data Fig. 4k). This result supports the notion that effector differentiation of anticancer CD8^+^ T cells occurs within tumour tissue. Unsupervised slingshot analysis of our TIL scRNA-seq data uncovered a tree-shaped developmental trajectory that begins with TCF1^+^ cells and progresses over CX_3_CR1^hi^TIM-3^+^ effector cells into CD7^hi^TIM-3^+^ effector cells, from which it branches off into distinct terminally differentiated T cell populations (Fig. 2e). Together, these results indicate a progressive trajectory for TIL differentiation within tumours that originates from TCF1^+^ TILs and follows a unidirectional path of effector differentiation before ending in multiple smaller branches of terminally differentiated TIL populations.

**Extended Data Fig. 5:**
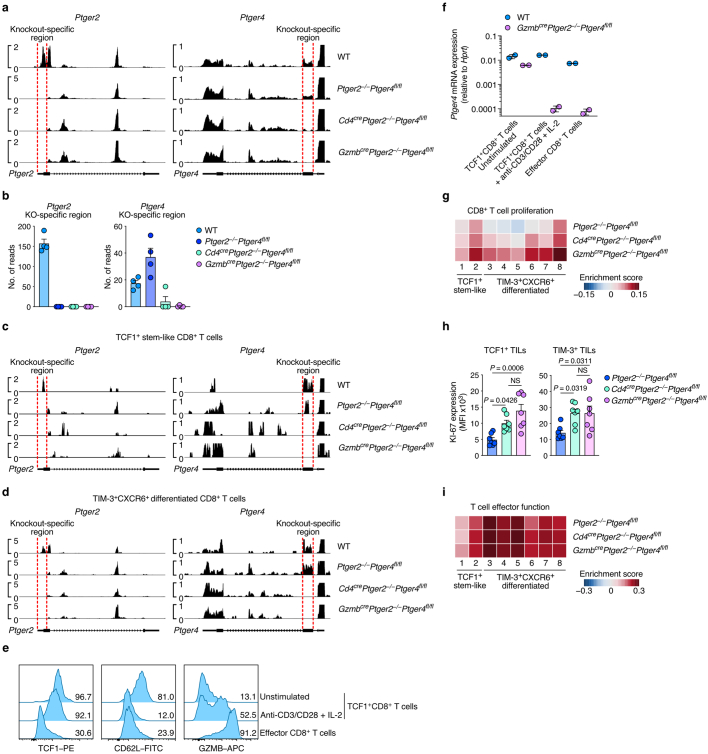
*Gzmb*^*cre*^*Ptger2*^*−/−*^*Ptger4*^*fl/fl*^ mice exhibit *Ptger4* knockout in TCF1^+^CD8^+^ TILs; increased proliferation of TCF1^+^CD8^+^ TILs and their progeny in T cell-specific EP_2_/EP_4_ double deficient mice. **a**, Gene tracks showing the average scRNA-seq read coverage on *Ptger2* and *Ptger4* loci in CD8^+^ TILs from BRAF^V600E^ melanoma tumours in WT, *Ptger2*^*−/−*^*Ptger4*^*fl/fl*^, *Cd4*^*cre*^*Ptger2*^*−/−*^*Ptger4*^*fl/fl*^ and *Gzmb*^*cre*^*Ptger2*^*−/−*^*Ptger4*^*fl/fl*^ mice across all replicates. Dashed boxes highlight knockout(KO)-specific target regions. **b**, Quantification of *Ptger2* and *Ptger4* read coverage for KO-specific target regions based on **a**. n = 4. **c**,**d**, Gene tracks showing the scRNA-seq read coverage on *Ptger2* and *Ptger4* loci in **c**, TCF1^+^CD8^+^ TILs and **d**, TIM-3^+^ TIL progeny from WT, *Ptger2*^*−/−*^*Ptger4*^*fl/fl*^, *Cd4*^*cre*^*Ptger2*^*−/−*^*Ptger4*^*fl/fl*^ and *Gzmb*^*cre*^*Ptger2*^*−/−*^*Ptger4*^*fl/fl*^ mice. Data for one representative replicate with comparable cell numbers is shown. **e**,**f**, WT or *Gzmb*^*cre*^*Ptger2*^−/−^*Ptger4*^*fl/fl*^ TCF1^+^ stem-like and TCF1^−^ effector CD8^+^ T cells from in vitro T cell cultures were stimulated or not with anti-CD3/CD28 for 24 h. **e**, Representative flow cytometry plots showing TCF1, CD62L and GZMB protein expression. **f**, *Ptger4* mRNA expression as measured by knockout-sensitive RT-PCR (n = 2). **g**, Expression of a CD8^+^ T cell proliferation signature across the distinct populations of CD8^+^ TILs from *Ptger2*^*−/−*^*Ptger4*^*fl/fl*^, *Cd4*^*cre*^*Ptger2*^*−/−*^*Ptger4*^*fl/fl*^ and *Gzmb*^*cre*^*Ptger2*^*−/−*^*Ptger4*^*fl/fl*^ mice based on scRNA-seq. **h**, Analysis of Ki-67 protein expression in TCF1^+^ and TIM-3^+^ CD8^+^ TILs 11 days after inoculation of *Ptger2*^*−/−*^*Ptger4*^*fl/fl*^, *Cd4*^*cre*^*Ptger2*^*−/−*^*Ptger4*^*fl/fl*^ and *Gzmb*^*cre*^*Ptger2*^*−/−*^*Ptger4*^*fl/fl*^ mice with 2 × 10^6^ control BRAF^V600E^ melanoma cells. n = 7. **i**, Expression of a gene signature for T cell effector function, analysed as in **g**. Data in **a**-**e**,**g**,**i** are from one experiment. Data in **b**,**h** are pooled from two (**b**) or three (**h**) independent experiments and depicted as mean ± s.e.m. Data in **f** are depicted as mean ± s.d. from one experiment. *P *values are from one-way ANOVA with Tukey’s multiple-comparison test. *P* ≥ 0.05, not significant (NS). Source Data

To assess the impact of PGE_2_–EP_2_/EP_4_ signalling on the landscape of CD8^+^ TILs, we separated our scRNA-seq data on the basis of recipient mouse groups. Density analysis revealed a prominent shift towards early effector (clusters 3 and 4) and terminally differentiated TIL populations (cluster 5) in both *Cd4*^*cre*^*Ptger2*^*−/−*^*Ptger4*^*fl/fl*^ mice and *Gzmb*^*cre*^*Ptger2*^*−/−*^*Ptger4*^*fl/fl*^ mice compared with *Ptger2*^*−/−*^*Ptger4*^*fl/fl*^ mice (Fig. 2f). We therefore quantified the frequencies TIL populations across all replicates. *Ptger2*^*−/−*^*Ptger4*^*fl/fl*^ mice lacked expansion of any differentiating effector TIL populations (Fig. 2g). By contrast, in *Cd4*^*cre*^*Ptger2*^*−/−*^*Ptger4*^*fl/fl*^ mice, we detected elevated frequencies of early and late effector TIL populations that further increased progressively along the common trajectory of effector differentiation (clusters 2–5; Fig. 2g). This pattern was similarly observed for *Gzmb*^*cre*^*Ptger2*^*−/−*^*Ptger4*^*fl/fl*^ mice (Fig. 2g), in which TIL expansion was even more prominent, which probably reflects additional favourable activity of intratumoural GZMB^+^ natural killer cells^15^. Enhanced TIL expansion in *Cd4*^*cre*^*Ptger2*^*−/−*^*Ptger4*^*fl/fl*^ mice and *Gzmb*^*cre*^*Ptger2*^*−/−*^*Ptger4*^*fl/fl*^ mice correlated with the fact that TCF1^+^ and TIM-3^+^CXCR6^+^ TILs in both models had lost *Ptger2* and *Ptger4* expression (Extended Data Fig. 5a–d). Consistent with the notion that intratumoural effector differentiation causes the loss of EP_4_ in TCF1^+^ TILs in *Gzmb*^*cre*^*Ptger2*^*−/−*^*Ptger4*^*fl/fl*^ mice, *Gzmb*^*cre*^*Ptger2*^*−/−*^*Ptger4*^*fl/f*^ TCF1^+^CD8^+^ T cells generated in vitro displayed efficient *Ptger4* ablation in an effector differentiation assay (Extended Data Fig. 5e,f). In line with enhanced TIL expansion, expression of a proliferation signature in effector TIL populations from *Cd4*^*cre*^*Ptger2*^*−/−*^*Ptger4*^*fl/fl*^ mice and *Gzmb*^*cre*^*Ptger2*^*−/−*^*Ptger4*^*fl/fl*^ mice was higher than from *Ptger2*^*−/−*^*Ptger4*^*fl/fl*^ mice (Extended Data Fig. 5g). Consistently, EP_2_/EP_4_-deficient TCF1^+^ TILs and their TIM-3^+^ progeny displayed increased expression of the proliferation marker Ki-67 (Extended Data Fig. 5h). However, we did not detect a substantial gain in expression of a gene signature for cytotoxic effector function in these TIL populations (Extended Data Fig. 5i). Thus, PGE_2_ does not modify the expression of genes associated with T cell function but prevents their differentiation and expansion, which highlights a mechanistic difference to canonical factors that drive dysfunctional CD8^+^ T cell responses through transcriptional programming, such as TOX^26,27^ or MYB^28^. Taken together, these findings suggest that tumour-derived PGE_2_ locally impairs the differentiation and expansion of effector T cell populations arising from TCF1^+^ TILs. Moreover, EP_2_/EP_4_ deficiency rescues TILs from this inhibitory effect of PGE_2_.

## PGE_2_–EP_2_/EP_4_ signalling limits clonal TIL expansion

**Extended Data Fig. 6:**
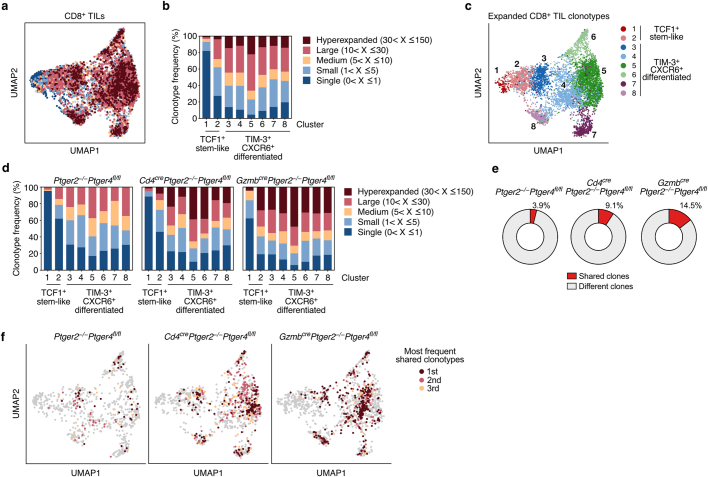
EP_2_/EP_4_ double deficiency permits clonal differentiation and expansion of CD8^+^ TILs in PGE_2_-producing tumours. **a**, UMAP visualisations of T cell clonotype distribution across CD8^+^ TILs based on scTCR-seq. **b**, Clonotype frequency across CD8^+^ TIL clusters based on **a**. **c**, UMAP visualisation of expanded tumour-infiltrating CD8^+^ T cell clonotypes coloured according to cluster classification. **d**, Comparison of clonotype frequencies between *Ptger2*^*−/−*^*Ptger4*^*fl/fl*^, *Cd4*^*cre*^*Ptger2*^*−/−*^*Ptger4*^*fl/fl*^ and *Gzmb*^*cre*^*Ptger2*^*−/−*^*Ptger4*^*fl/fl*^ mice. **e**, Frequencies of T cell clones shared between TCF1^+^CD8^+^ TILs and TIM-3^+^ TIL progeny. **f**, UMAP plots visualising the top 3 most frequent shared clonotypes for *Ptger2*^*−/−*^*Ptger4*^*fl/fl*^, *Cd4*^*cre*^*Ptger2*^*−/−*^*Ptger4*^*fl/fl*^ and *Gzmb*^*cre*^*Ptger2*^*−/−*^*Ptger4*^*fl/fl*^ mice. Data are from one experiment. Source Data

Analyses of our scTCR-seq data revealed that many CD8^+^ TILs consisted of clonally expanded cells (Extended Data Fig. 6a–c), which is an indicator of tumour specificity and proliferative T cell expansion^29,30^. Notably, although expanded clonotypes were detectable in all TIL populations, they were more prominent among TIM-3^+^CXCR6^+^ cells (clusters 3–8) (Extended Data Fig. 6a–c), a result consistent with the notion that these differentiated TIL populations may arise locally through the proliferative expansion of a few tumour-specific TCF1^+^ TILs. Moreover, highly expanded TIL clones (>30) were completely absent from tumours in *Ptger2*^*−/−*^*Ptger4*^*fl/fl*^ mice but were abundant in both *Cd4*^*cre*^*Ptger2*^*−/−*^*Ptger4*^*fl/fl*^ mice (22.6%) and *Gzmb*^*cre*^*Ptger2*^*−/−*^*Ptger4*^*fl/fl*^ mice (35.6%) (Fig. 2h,i and Extended Data Fig. 6d). Consistently, *Ptger2*^*−/−*^*Ptger4*^*fl/fl*^ mice displayed an increased frequency of poorly expanded small or single clones (Fig. 2h,i and Extended Data Fig. 6d). Notably, TILs in both *Cd4*^*cre*^*Ptger2*^*−/−*^*Ptger4*^*fl/fl*^ mice and *Gzmb*^*cre*^*Ptger2*^*−/−*^*Ptger4*^*fl/fl*^ mice had much higher fractions of clones shared between TCF1^+^ cells and TIM-3^+^CXCR6^+^ effector progeny (9.1% and 14.5%, respectively) than *Ptger2*^*−/−*^*Ptger4*^*fl/fl*^ mice (3.9%) (Extended Data Fig. 6e). The few shared clones detectable in *Ptger2*^*−/−*^*Ptger4*^*fl/fl*^ mice, however, only showed poor expansion (Extended Data Fig. 6f). We conclude that tumour-derived PGE_2_ restricts clonal TIL expansion, which results in a collapse of the intratumoural CD8^+^ T cell response. This impairment is overcome by ablation of EP_2_/EP_4_ in CD8^+^ T cells, which leads to the productive differentiation and expansion of clonal effector T cell progeny within tumour tissue.

## TCF1^+^ TIL effector expansion achieves tumour control

**Extended Data Fig. 7:**
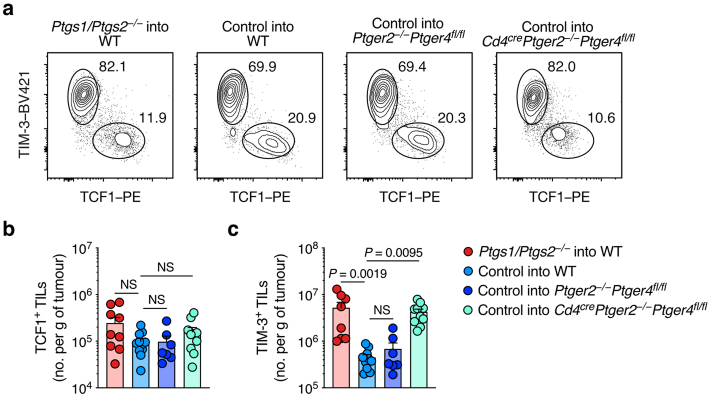
EP_2_/EP_4_ double deficiency rescues development of early and terminally differentiated effector CD8^+^ T cells within tumour tissue. **a**-**c**, WT, *Ptger2*^*−/−*^*Ptger4*^*fl/fl*^ or *Cd4*^*cre*^*Ptger2*^*−/−*^*Ptger4*^*fl/fl*^ mice were injected s.c. with 2×10^6^ control or *Ptgs1/Ptgs2*^*−/−*^ BRAF^V600E^ cells and CD44^+^ CD8^+^ TILs were analysed 11 days later by flow cytometry. **a**, Representative plots showing the frequency of TCF1^+^ stem-like and TIM-3^+^ effector CD8^+^ TIL populations. **b**, Quantification of TCF1^+^CD8^+^ TILs (*Ptgs1/Ptgs2*^−/−^ into WT, n = 9; Control into WT, n = 11; Control into *Ptger2*^*−/−*^*Ptger4*^*fl/fl*^, n = 7; Control into *Cd4*^*cre*^*Ptger2*^*−/−*^*Ptger4*^*fl/fl*^, n = 10). **c**, Quantification of TIM-3^+^CD8^+^ TILs (*Ptgs1/Ptgs2*^−/−^ into WT, n = 8; Control into WT, n = 10; Control into *Ptger2*^*−/−*^*Ptger4*^*fl/fl*^, n = 7; Control into *Cd4*^*cre*^*Ptger2*^*−/−*^*Ptger4*^*fl/fl*^, n = 10). Data in **b**,**c** are pooled from three independent experiments and depicted as mean ± s.e.m. Plots in **a** show data for one tumour sample representative for at least n = 7 samples from three independent experiments. *P *values are from one-way ANOVA with Tukey’s multiple-comparison test. *P* ≥ 0.05, not significant (NS). Source Data

We next sought to provide further evidence that EP_2_/EP_4_ deficiency in CD8^+^ T cells permits productive effector differentiation of TILs in PGE_2_-producing tumours. Quantification of TIL populations across PGE_2_-deficient *Ptgs1/Ptgs2*^*−/−*^ BRAF^V600E^ tumours from WT mice and PGE_2_-producing control BRAF^V600E^ from WT mice, *Ptger2*^*−/−*^*Ptger4*^*fl/fl*^ mice and *Cd4*^*cre*^*Ptger2*^*−/−*^*Ptger4*^*fl/fl*^ mice (Extended Data Fig. 7a–c) revealed that the numbers of TCF1^+^ TILs were comparable among all groups (Extended Data Fig. 7b). This result indicated that the generation of TCF1^+^CD8^+^ T cells in lymphoid tissues and their tumour infiltration is not affected by PGE_2_. However, whereas the numbers of differentiated TIM-3^+^ TILs were low in control BRAF^V600E^ tumours in WT mice and *Ptger2*^*−/−*^*Ptger4*^*fl/fl*^ mice, they were highly abundant in *Cd4*^*cre*^*Ptger2*^*−/−*^*Ptger4*^*fl/fl*^ mice (Extended Data Fig. 7c) and indistinguishable from those found in *Ptgs1/Ptgs2*^*−/−*^ BRAF^V600E^ tumours in WT mice.

To determine whether TIM-3^+^ TILs were generated from TCF1^+^ TILs locally within tumour tissue, we made use of our finding that at early stages after implantation (day 6), tumours contain TCF1^+^ TILs but not (yet) differentiated TIM-3^+^ TILs (Fig. 2j–n). Tumour-bearing *Cd4*^*cre*^*Ptger2*^*−/−*^*Ptger4*^*fl/fl*^ mice treated from day 6 onwards with the S1P1R antagonist FTY720, which prevents lymph node (LN) egress of newly primed CD8^+^ T cells^31^, showed unabated intratumoural development and prominent expansion of TIM-3^+^ TILs over time (Fig. 2k–m). By contrast, when initial tumour infiltration of TCF1^+^CD8^+^ T cells was blocked by FTY720 application from day 1 onwards, no intratumoural TIL expansion was detected (Fig. 2m). Notably, the proliferative response originating from TCF1^+^ TILs present in tumour tissue at day 6 was sufficient to achieve control of tumour growth (Fig. 2n). This result demonstrates that TCF1^+^ TILs locally generate potent anticancer effector responses when protected from inhibitory PGE_2_ signalling in tumours.

## PGE_2_ suppresses IL-2 responsiveness of TILs

**Fig. 3:**
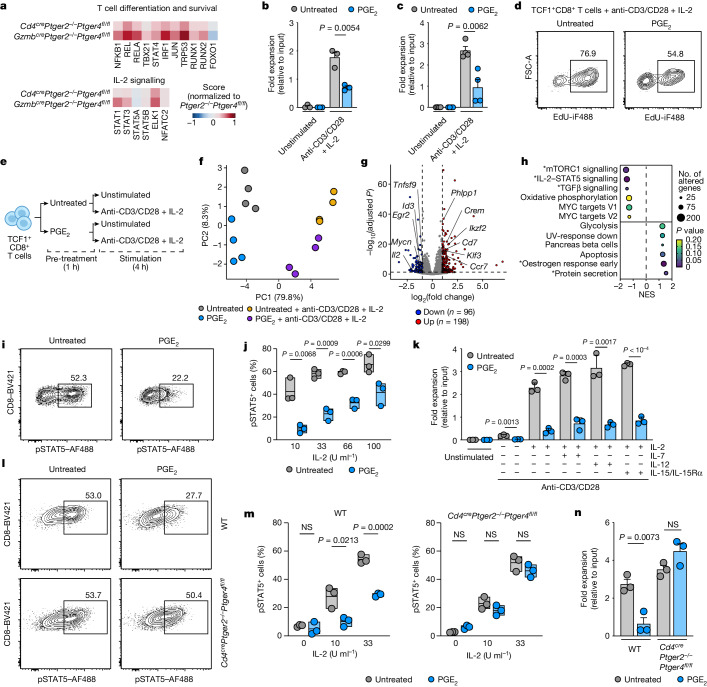
PGE_2_ impairs CD8^+^ T cell expansion and effector differentiation from TCF1^+^ cells by inhibiting IL-2 signalling. **a**, TF activity in TCF1^+^CD8^+^ TILs from control BRAF^V600E^ tumours in *Cd4*^*cre*^*Ptger2*^*−/−*^*Ptger4*^*fl/fl*^ mice and *Gzmb*^*cre*^*Ptger2*^*−/−*^*Ptger4*^*fl/fl*^ mice (relative to *Ptger2*^*−/−*^*Ptger4*^*fl/fl*^ mice). **b**, Effect of PGE_2_ on ex vivo expansion of TCF1^+^CD8^+^ TILs sorted from *Ptgs1/Ptgs2*^*−/−*^ BRAF^V600E^ tumours (*n* = 3). **c**,**d**, Effect of PGE_2_ on expansion (**c**) and proliferation (**d**) of repetitively activated TCF1^+^CD8^+^ T cells from in vitro T cell cultures (*n* = 4). **e**–**h**, Analysis of repetitively activated TCF1^+^CD8^+^ T cells by RNA-seq (*n* = 4). **e**, Experimental design. **f**, principal component (PC) analysis based on all DEGs. **g**, Volcano plot showing the effect of PGE_2_ exposure on gene expression in TCF1^+^CD8^+^ T cells stimulated with anti-CD3/CD28 and IL-2. **h**, GSEA of hallmark pathways based on **g**. *Pathways significantly regulated; NES, normalized enrichment score. **i**,**j**, Effect of PGE_2_ exposure on IL-2-dependent pSTAT5 induction in repetitively activated TCF1^+^CD8^+^ T cells. Cells were treated with 33 U ml^–1^ IL-2. **j**, *n* = 3. **k**, Expansion of repetitively activated TCF1^+^CD8^+^ T cells treated or untreated with PGE_2_ and stimulated as indicated (*n* = 3). **l**–**n**, WT or *Cd4*^*cre*^*Ptger2*^−/−^*Ptger4*^*fl/fl*^ TCF1^+^CD8^+^ T cells from in vitro T cell cultures were incubated with or without PGE_2_ for 20 h before stimulation with IL-2 (**l**,**m**) or anti-CD3/CD28 and IL-2 (**n**). **l**, Flow cytometry plot showing pSTAT5 signalling after 30 min. Cells were treated with 33 U ml^–1^ IL-2. **m**, Quantification of pSTAT5 (*n* = 3). **n**, Quantification of T cell expansion (*n* = 3). Data in **b** and **c** are pooled from two independent experiments. Data in **j**, **k**, **m** and **n** are representative of two independent experiments. Plots in **d**, **i** and **l** show data for 1 T cell culture representative of *n* = 6 T cell cultures analysed in 2 independent experiments. For **b**, **c**, **k** and **n**, horizontal lines and error bars indicate the mean ± s.e.m. For **j** and **m**, box plots indicate the median. *P* values are from unpaired *t*-tests. In **g**, DEGs (*P* < 0.05; fold change ≥ 2) were identified by Wald test with multiple testing using the Benjamini–Hochberg method. Source Data

**Extended Data Fig. 8:**
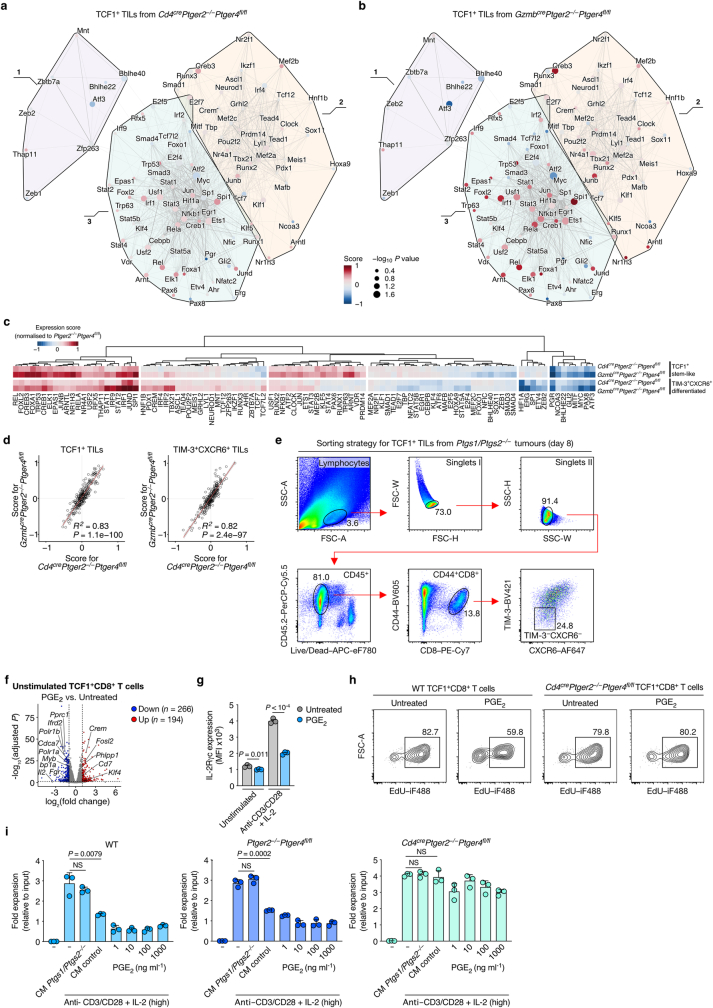
PGE_2_-mediated changes in TCF1^+^CD8^+^ T cells, effect on γc expression and proliferation. **a**-**d**, scRNA-seq based analysis of TF activity alterations in TCF1^+^CD8^+^ TILs and their TIM-3^+^CXCR6^+^ effector progeny between control BRAF^V600E^ melanoma tumours in *Cd4*^*cre*^*Ptger2*^*−/−*^*Ptger4*^*fl/fl*^ and *Gzmb*^*cre*^*Ptger2*^*−/−*^*Ptger4*^*fl/fl*^ mice compared to *Ptger2*^*−/−*^*Ptger4*^*fl/fl*^ mice. **a**,**b**, Alterations of TF network activity in TCF1^+^CD8^+^ TILs from BRAF^V600E^ melanoma tumours in **a**, *Cd4*^*cre*^*Ptger2*^*−/−*^*Ptger4*^*fl/fl*^ mice compared to *Ptger2*^*−/−*^*Ptger4*^*fl/fl*^ mice or **b**, *Gzmb*^*cre*^*Ptger2*^*−/−*^*Ptger4*^*fl/fl*^ mice compared to *Ptger2*^*−/−*^*Ptger4*^*fl/fl*^ mice. **c**, Heatmap visualisation, **d**, Correlation of TF activity alterations for TIL populations from *Cd4*^*cre*^*Ptger2*^*−/−*^*Ptger4*^*fl/fl*^ and *GzmbB*^*cre*^*Ptger2*^*−/−*^*Ptger4*^*fl/fl*^ mice. **e**, Flow cytometry plots showing the sorting strategy for TCF1^+^ TILs (identified as TIM-3^−^CXCR6^−^ cells) from *Ptgs1/Ptgs2*^*−/−*^ BRAF^V600E^ tumours. **f**, Volcano plot showing the effect of PGE_2_ exposure on gene expression in TCF1^+^CD8^+^ T cells from in vitro T cell cultures, based on RNA-seq analysis. **g**, Effect of PGE_2_ treatment on IL-2Rγc protein expression (n = 3). **h**, WT or *Cd4*^*cre*^*Ptger2*^−/−^*Ptger4*^*fl/fl*^ TCF1^+^CD8^+^ T cells from in vitro T cell cultures were stimulated with anti-CD3/CD28 and high-dose IL-2 in presence or absence of PGE_2_. After 24 h cells were analysed for T cell proliferation by EdU incorporation. **i**, WT, *Ptger2*^−/−^*Ptger4*^*fl/fl*^ or *Cd4*^*cre*^*Ptger2*^−/−^*Ptger4*^*fl/fl*^ TCF1^+^CD8^+^ T cells were treated or not with tumour cell conditioned medium (CM) or PGE_2_ and stimulated as indicated. After 72 h, T cell expansion was analysed by flow cytometry. n = 3. Data in **a**-**d**, **f** are from one experiment. Data in **g**,**i** show data for one representative of two independent experiments with n = 3 biological replicates. In **f**), DEGs (*P* < 0.05; fold change ≥ 2) were identified by Wald test with multiple testing by the Benjamini-Hochberg method. Horizontal lines and error bars in (**g**,**i**) indicate mean ± s.d. *P-*values are from **d**, Fitted linear regression models or **g**, and **i**, unpaired t-test. *P* ≥ 0.05, not significant (NS). Plots in (**h**) show data for one sample representative for n = 3 samples. Source Data

In an effort to identify the mechanisms downstream of PGE_2_–EP_2_/EP_4_ signalling that determine impaired TIL responses, we performed TF activity analysis of our scRNA-seq data. Deficiency of EP_2_/EP_4_ in TCF1^+^ TILs resulted in an increased activity of TFs associated with effector differentiation (including NFKB1, REL, JUN and TBX21), stimulatory cytokine signalling (STAT4, IRF1, NFKB1, JUN and TBX21) and survival (RUNX2 and TRP53) (Fig. 3a and Extended Data Fig. 8a,b). Most of these alterations were detectable in both TCF1^+^ TILs and their developing progeny (Extended Data Fig. 8c) and were highly consistent across *Cd4*^*cre*^*Ptger2*^*−/−*^*Ptger4*^*fl/fl*^ mice and *Gzmb*^*cre*^*Ptger2*^*−/−*^*Ptger4*^*fl/fl*^ mice (Fig. 3a and Extended Data Fig. 8c,d). Notably, we detected increased TF activity linked to IL-2 cytokine signalling (including STAT1, STAT3, STAT5B, ELK1 and NFATC2)^32^ in TILs from *Cd4*^*cre*^*Ptger2*^*−/−*^*Ptger4*^*fl/fl*^ mice and *Gzmb*^*cre*^*Ptger2*^*−/−*^*Ptger4*^*fl/fl*^ mice (Fig. 3a and Extended Data Fig. 8a,b). This result was again observed in both TCF1^+^ TILs and their TIM-3^+^CXCR6^+^ progeny (Extended Data Fig. 8c,d). PGE_2_ may therefore affect the response of TILs to IL-2, which is a notable finding given the current development of new classes of IL-2 receptor (IL-2R) agonists for cancer therapy and the emerging role of IL-2 signalling for productive anticancer responses by TCF1^+^ TILs^33,34^.

We therefore tested whether PGE_2_ controls the IL-2-mediated expansion of TCF1^+^ TILs sorted from PGE_2_-deficient *Ptgs1/Ptgs2*^*−/−*^ tumours (identified as TIM-3^−^CXCR6^−^ TILs; Extended Data Fig. 8e). PGE_2_ strongly compromised the capacity of TCF1^+^ TILs to expand and differentiate into effector cells when stimulated with high-dose IL-2 together with anti-CD3 and anti-CD28 (anti-CD3/CD28) treatment^35,36^ (Fig. 3b). Bypassing the scarcity of TIL numbers, we further addressed this issue using antigen-experienced, repetitively activated TCF1^+^CD8^+^ T  cells generated in vitro, on which PGE_2_ had an identical inhibitory effect (Fig. 3c). In line with PGE_2_-mediated impairment of IL-2-driven proliferation and effector differentiation, PGE_2_-treated TCF1^+^CD8^+^ T cells from in vitro T cell cultures showed markedly reduced DNA replication early after their stimulation (Fig 3d). Transcriptional profiling by RNA-seq (Fig. 3e) revealed that PGE_2_ exposure resulted in distinct transcriptional changes in stimulated TCF1^+^CD8^+^ T cells and their unstimulated counterparts (Fig. 3f). Analyses of the stimulated T cell populations identified 294 differentially expressed genes (DEGs) following PGE_2_ exposure (Fig 3g). PGE_2_-treated TCF1^+^CD8^+^ T cell populations expressed increased levels of transcripts encoding for molecules related to EP_2_/EP_4_-mediated cAMP signalling (*Crem* and *Fosl2*) and T cell quiescence (for example, *Phlpp1, Klf3* and *Klf4*) (Fig. 3g and Extended Data Fig. 8f). Gene set enrichment analysis (GSEA) showed a selective downregulation of the T cell differentiation-associated mTORC1 signalling pathway and the IL-2 signalling pathway (Fig. 3h). The latter result is consistent with the observed reduced IL-2 pathway activity in TILs identified in our scRNA-seq analysis and further supports the notion that PGE_2_ impairs the proliferative expansion of TILs through the inhibition of IL-2 signalling.

In line with an inhibitory effect of PGE_2_ on IL-2 signalling, IL-2 stimulation failed to promote STAT5 phosphorylation (pSTAT5) in PGE_2_-treated TCF1^+^CD8^+^ T cells (Fig. 3i). Notably, this defect was associated with reduced surface expression of the IL-2R gamma chain (IL-2Rγc) (Extended Data Fig. 8g) and could only partially be rescued by stimulation with high doses of IL-2 (Fig. 3j). This result points towards a dominant inhibitory effect of PGE_2_ on IL-2 signalling through the regulation of IL-2Rγc expression. Consistent with this notion, PGE_2_ impaired the expansion of TCF1^+^CD8^+^ T cells not only in response to IL-2 but also the γc cytokines IL-7 and IL-15 (Fig. 3k). Thus, PGE_2_ fundamentally affects the entire γc cytokine signalling pathway in TCF1^+^CD8^+^ T cells and their differentiating progeny. IL-2-mediated pSTAT5 (Fig. 3l,m) and IL-2-dependent T cell proliferation and expansion (Fig. 3n and Extended Data Fig. 8h,i) was rescued in TCF1^+^CD8^+^ T cells from *Cd4*^*cre*^*Ptger2*^*−/−*^*Ptger4*^*fl/fl*^ mice, which demonstrates the functional relevance of EP_2_/EP_4_ signalling for the restriction of IL-2 signalling. Taken together, these data suggest that the PGE_2_–EP_2_/EP_4_ axis limits productive anticancer TIL responses by suppressing the IL-2 signalling pathway.

## EP_2_/EP_4_-deficient TILs mediate cancer elimination

**Fig. 4:**
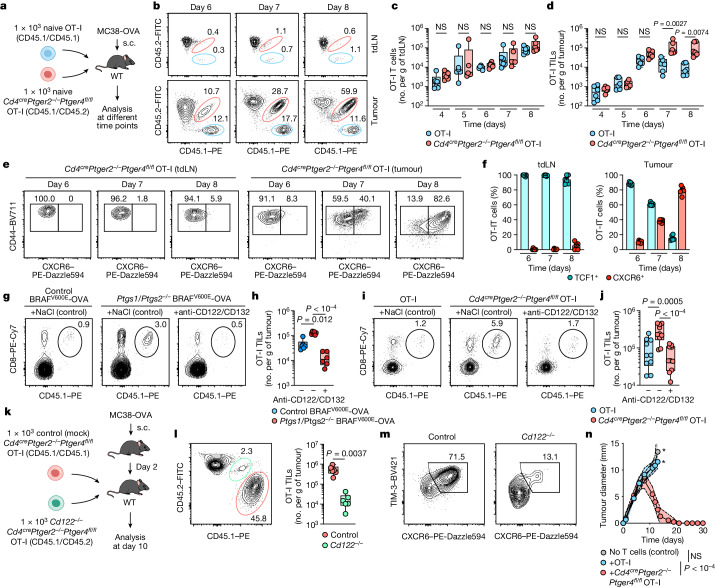
EP_2_/EP_4_-deficient tumour antigen-specific CD8^+^ T cells expand in PGE_2_-producing tumours and mediate tumour immune control. **a**, Experimental design for **b**–**f**. **b**, Flow cytometric plots of CD8^+^ T cells from tdLNs and tumours from the indicated days. **c**,**d**, Numbers of expanded OT-I CD8^+^ T cells in tdLNs (**c**) and tumours (**d**) at indicated time points (*n* = 6). **e**,**f**, Analysis of CD44 and CXCR6 expression in *Cd4*^*cre*^*Ptger2*^−/−^*Ptger4*^*fl/fl*^ OT-I cells. **e**, Flow cytometry plots. **f**, Subset frequencies (*n* = 6). **g**–**j**, Effect of CD122/CD132 blockade on OT-I T cell expansion in tumours. **g**–**j**, Effect of anti-CD122 and anti-CD132 (anti-CD122/CD132) treatment on OT-I TIL expansion in WT mice with control or *Ptgs1/Ptgs2*^−/−^ BRAF^V600E^-OVA tumours or with MC38-OVA tumours, analysed 11 days after tumour transplantation. **g**,**h**, Flow cytometry plots (**g**) and OT-I TIL numbers (**h**) in BRAF^V600E^-OVA tumours (*n* = 6). **i**,**j**, Flow cytometry plots (**i**) and OT-I TIL numbers (**j**) in MC38-OVA tumours (*n* = 10). **k**, Experimental design for **l** and **m** with MC38-OVA tumours. **l**, Flow cytometry plot (left) and quantification (right) of OT-I TILs at day 10 (*n* = 6). **m**, Flow cytometry plots showing the population size of TIM-3^+^CXCR6^+^ cells among control and *Cd122*^*−/−*^ *Cd4*^*cre*^*Ptger2*^*−/−*^*Ptger4*^*fl/fl*^ OT-I TILs. **n**, WT mice received 1 × 10^3^ naive OT-I T cells or 1 × 10^3^ naive *Cd4*^*c*^^*re*^*Ptger2*^−/−^*Ptger4*^*fl/fl*^ OT-I T cells intravenously (i.v.) and were transplanted s.c. with 2 × 10^6^ MC38-OVA cells before analysis of tumour growth over time (*n* = 10). Asterisk indicates that termination criteria were reached. Data in **c**, **d**, **f**, **h**, **j**, **l** and **n** are pooled from two (**c**,**d**,**h**,**l**) or three (**f**,**j**,**n**) independent experiments and depicted as box plots extending from the 25th to 75th percentiles with the median as the centre and the whiskers corresponding to minimum and maximum values (**c**,**d**,**h**,**j**,**l**) or shown as the mean ± s.e.m. (**f**,**n**). Plots in **b**, **e**, **g**, **i**, **l** and **m** show data for 1 sample representative of *n* = 6 samples analysed in 2 (**b**,**g**,**l**,**m**) or 3 (**e**,**i**) independent experiments. *P* values are from paired *t*-tests (**l**), one-way ANOVA with Tukey’s multiple-comparison test (**c**,**d**) or Dunnett’s multiple-comparison test (**h**,**j**), or two-way ANOVA with Bonferroni’s correction for multiple testing (**n**). Source Data

**Extended Data Fig. 9:**
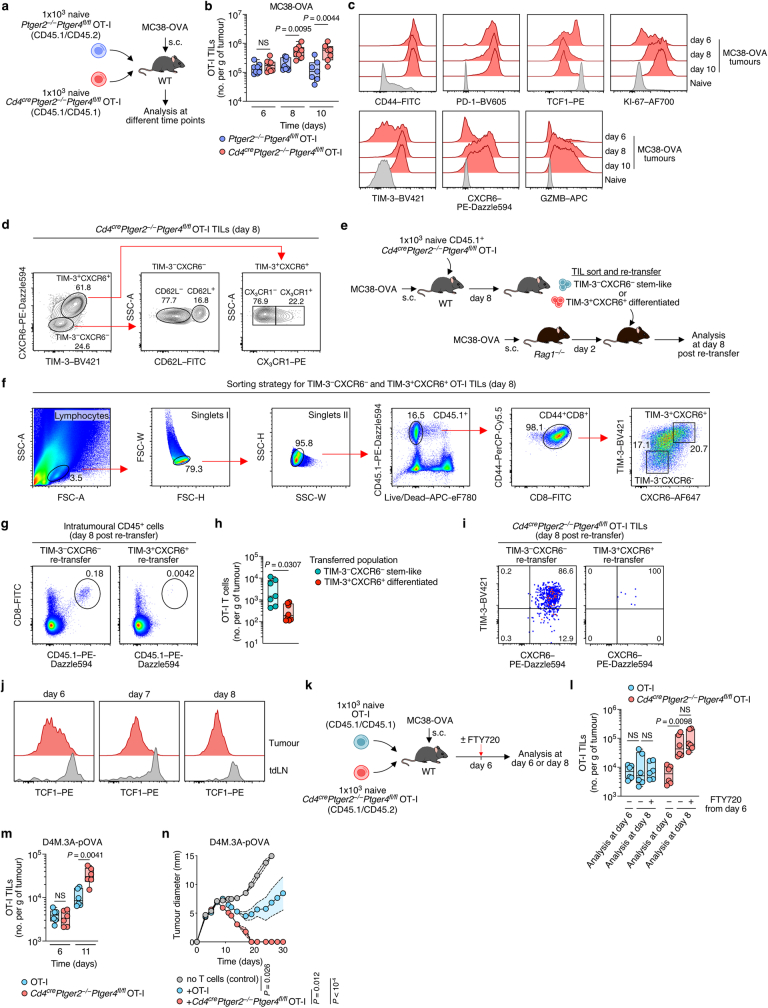
PGE_2_ impairs proliferative expansion and effector differentiation of antigen-specific TCF1^+^CD8^+^ TILs. **a**, Experimental design for **b**-**d** **b**, Quantification of OT-I cell expansion in tumours at different time points (day 6 and day 8: n = 8 per group; day 10: n = 7 per group). **c**, Histograms showing expression of indicated molecules in *Cd4*^*cre*^*Ptger2*^−/−^*Ptger4*^*fl/fl*^ OT-I TILs at different time points. Naive *Cd4*^*cre*^*Ptger2*^−/−^*Ptger4*^*fl/fl*^ OT-I T cells served as control. **d**, Flow cytometry plots showing subpopulation composition among *Cd4*^*cre*^*Ptger2*^−/−^*Ptger4*^*fl/fl*^ OT-I TILs. **e-h**, WT mice received 1 × 10^3^ naive congenically marked CD45.1^+^ *Cd4*^*cre*^*Ptger2*^−/−^*Ptger4*^*fl/fl*^ OT-I T cells followed by inoculation with 2 × 10^6^ MC38-OVA cells. 8 days later, the indicated subpopulations of OT-I TILs were sorted and 7 × 10^3^ cells were re-transferred into MC38-OVA bearing *Rag1*^−/−^ recipient mice. TILs in *Rag1*^−/−^ recipients were analysed at day 8 post re-transfer. **e**, Experimental design. **f**, Flow cytometry plots showing the sorting strategy. **g**, Flow cytometry plots showing the frequency of OT-I TILs recovered at day 8 post re-transfer. **h**, Quantification of OT-I TIL progenies based on (**g**), with TIM-3^−^CXCR6^−^ stem-like, n = 7; TIM-3^+^CXCR6^+^ differentiated, n = 8. **i**, Flow cytometry plots showing the expression of TIM-3 and CXCR6 among recovered OT-I TILs. **j**, Analysis of TCF1 expression in *Cd4*^*cre*^*Ptger2*^−/−^*Ptger4*^*fl/fl*^ OT-I T cells in tdLNs and tumours over time. **k**, Experimental design for **l**. **l**, Effect of FTY720 treatment from day 6 on OT-I T cell expansion in tumours (n = 6). **m**,**n**, WT mice received 1 × 10^3^ naive OT-I T cells or 1 × 10^3^ naive *Cd4*^*cre*^*Ptger2*^−/−^*Ptger4*^*fl/fl*^ OT-I T cells i.v. and were transplanted s.c. with 2 × 10^6^ D4M.3A-pOVA melanoma cells before **m**, quantification of OT-I cell expansion in tumours at different time points (n = 6) or **n**, analysis of tumour growth over time (n = 4). Data in **b**,**h**,**l**-**n** are pooled from two (**h**,**l**-**n**) or three (**b**) independent experiments and depicted as box plots extending from the 25^th^ to 75^th^ percentiles with the median as centre and whiskers corresponding to minimum and maximum values (**b**,**h**,**l**,**m**) or shown as mean ± s.e.m. (**n**). Plots in **c**,**d**,**g**,**i**,**j** show data for one sample representative for (**c**,**d**,**g**,**i**) n = 7 or (**j**) n = 6 samples from two independent experiments. *P *values in **b,l** are from one-way ANOVA with Tukey’s multiple-comparison test, *P *values in **h**,**m** are from unpaired t-test, *P-*values in **n** are from two-way ANOVA with Bonferroni’s correction for multiple testing. *P* ≥ 0.05, not significant (NS). Source Data

To examine antigen-specific TIL responses in more detail, we used WT (EP_2_/EP_4_-proficient) and *Cd4*^*cre*^*Ptger2*^*−/−*^*Ptger4*^*fl/fl*^ (EP_2_/EP_4_-deficient) OT-I T cells. We co-transferred a small number (1 × 10^3^ cells) of congenically marked WT and EP_2_/EP_4_-deficient OT-I T cells into recipient mice, which were subsequently challenged with MC38-OVA tumours (Fig. 4a). Consistent with the observation that PGE_2_ selectively inhibits CD8^+^ T cells within tumours, both WT and EP_2_/EP_4_-deficient OT-I T cells displayed prominent and unrestricted expansion in tdLNs (Fig. 4b,c). However, whereas the response by WT OT-I T cells collapsed after the initial phase of tumour infiltration (Fig. 4b,d), EP_2_/EP_4_-deficient OT-I T cells underwent persistent expansion in tumour tissue (Fig. 4b,d). Similarly, EP_2_-deficient OT-I T cells showed inefficient intratumoural expansion compared with EP_2_/EP_4_-deficient OT-I T cells (Extended Data Fig. 9a,b). Notably, EP_2_/EP_4_-deficient TCF1^+^ OT-I TILs over time gave rise to phenotypically distinct populations of TIM-3^+^CXCR6^+^ effector cells (Extended Data Fig. 9c,d). Re-transfer experiments (Extended Data Fig. 9e,f) confirmed that EP_2_/EP_4_-deficient TIM-3^−^(TCF1^+^) OT-I TILs but not their TIM-3^+^(TCF1^−^) descendants possessed the capacity to expand in tumours (Extended Data Fig. 9g,h) and were able to give rise to TIM-3^+^CXCR6^+^ TILs (Extended Data Fig. 9i). In separate experiments, we also observed that the development of TIM-3^+^ effector progeny from TCF1^+^ EP_2_/EP_4_-deficient OT-I T cells exclusively occurred in tumours but not in tdLNs (Fig. 4e,f and Extended Data Fig. 9j). Together, these data suggest that clonal T cell effector differentiation is restricted to tumour tissue and originates from TCF1^+^ TILs^1–3^. Consistent with this notion, and similar to our observations for polyclonal anticancer CD8^+^ T cell responses, FTY720-mediated blockade of T cell egress from LNs from day 6 onwards had no impact on the local expansion of EP_2_/EP_4_-deficient OT-I TILs (Extended Data Fig. 9k,l). We conclude that tumour-specific TCF1^+^ TILs expand and give rise to effector progeny within tumours, and this pivotal phase of the anticancer CD8^+^ T cell responses is blunted by PGE_2_.

In vivo blockade of IL-2R signalling using blocking antibodies against the IL-2Rβ (also known as CD122) and IL-2Rγc (also known as CD132) chains abrogated the expansion advantage of OT-I TILs in PGE_2_-deficient *Ptgs1/Ptgs2*^*−/−*^ BRAF^V600E^-OVA tumours (Fig. 4g,h) and that of EP_2_/EP_4_-deficient OT-I TILs in PGE_2_-producing MC38-OVA tumours (Fig. 4i,j). Similar results were observed after T cell-specific ablation of IL-2Rβ expression (Fig. 4k), which resulted in markedly reduced expansion (Fig. 4l) and effector differentiation (Fig. 4m) of EP_2_/EP_4_-deficient OT-I TILs compared with mock-treated control EP_2_/EP_4_-deficient OT-I TILs. Therefore, the IL-2R signalling pathway drives the expansion and effector differentiation of antigen-specific CD8^+^ TILs in the absence of PGE_2_–EP_2_/EP_4_ signalling.

Finally, to specifically evaluate whether EP_2_/EP_4_-deficient antigen-specific CD8^+^ T cells mount protective anticancer responses, we analysed the growth of MC38-OVA tumours transplanted into WT mice with or without transfer of WT or EP_2_/EP_4_-deficient OT-I T cells. EP_2_/EP_4_-deficient OT-I T cells achieved complete rejection of MC38-OVA tumours, whereas WT OT-I T cells failed to affect progressive MC38-OVA tumour growth (Fig. 4n). Of note, EP_2_/EP_4_-deficient OT-I but not WT OT-I TILs also showed enhanced expansion that led to efficient tumour elimination in mouse melanoma D4M.3A-pOVA tumours (Extended Data Fig. 9m,n). Taken together, these results suggest that interfering with the PGE_2_–EP_2_/EP_4_ axis in cancer-specific CD8^+^ T cells can elicit their expansion and effector differentiation within tumours and result in protective T cell-mediated anticancer immunity.

## Discussion

Our results demonstrate that tumour-derived PGE_2_ acts locally within the tumour microenvironment to limit CD8^+^ TIL expansion and effector differentiation originating from TCF1^+^ stem-like TILs. This inhibitory mechanism is crucial for cancer immune escape. We reveal that PGE_2_-mediated restriction of TIL responses generated from TCF1^+^ TILs depends on TIL-intrinsic signalling of the PGE_2_-receptors EP_2_ and EP_4_, which causes downregulation of functional IL-2 receptors and curtails TIL responsiveness to IL-2. As a result, interference with PGE_2_–EP_2_/EP_4_ signalling in CD8^+^ T cells enhances their IL-2 responsiveness and induces protective TIL-mediated anticancer immunity. Of note, the effect of PGE_2_ on TIL expansion and effector differentiation may at least in part be linked to a defect in IL-2-dependent mTORC1 signalling, as also suggested by an accompanying paper^37^.

Beyond highlighting that clonal expansion and effector differentiation of stem-like TCF1^+^CD8^+^ T cells occurs within tumour tissue, as recently suggested^1–3^, our results reveal that this critical phase of protective anticancer immunity is selectively targeted by tumour-derived PGE_2_. These findings therefore identify an intratumoural checkpoint that locally controls expansion and effector differentiation of cancer-specific CD8^+^ TILs. Of note, this mechanism may act in parallel to PGE_2_-mediated inhibition of cDC1 (ref. ^38^), which can support TCF1^+^ TIL responses within the tumour microenvironment^39^.

Our unbiased transcriptional profiling by scRNA-seq uncovered that protective anticancer responses by EP_2_/EP_4_-deficient TILs are coupled to a rescue of IL-2 signalling. Recent studies have highlighted the relevance of IL-2 signalling for the generation of effective CD8^+^ T cell responses from antigen-specific TCF1^+^CD8^+^ T cells^6,34,40,41^. Therefore, the discovery that the PGE_2_–EP_2_/EP_4_ axis antagonizes the responsiveness of TCF1^+^ TILs to IL-2 has important mechanistic and clinical implications. Our results provide evidence that PGE_2_ limits the proliferative capacity (and hence likely the self-renewal) of TCF1^+^ stem-like TILs and at the same time curbs effector T cell generation along the entire pathway of intratumoural TIL differentiation. Importantly, ablation of PGE_2_ signalling and consequently reconstitution of IL-2 signalling sufficed to achieve clonal TIL expansion and their effector differentiation within tumours that was not accompanied while preserving TCF1^+^ stem-like TILs. This is fundamentally different to interfering with exhaustion-inducing transcription factors such as TOX or MYB, which comes at the expense of TCF1^+^ stem-like CD8^+^ T cells and leads to a substantial change towards the development of terminally differentiated dysfunctional T cells^26–28^. Moreover, our finding that abrogating PGE_2_ signalling in T cells enhances clonal expansion across the entire differentiation spectrum of anticancer TILs indicates that physiological IL-2 concentrations within tumours are sufficient to drive protective anticancer immunity if IL-2 signalling in TILs is restored.

Targeting EP_2_ and EP_4_ on anticancer T cells to overcome PGE_2_-induced curtailing of IL-2 responsiveness might be preferential over using high concentrations of IL-2, as the latter may lead to deleterious off-target effects of IL-2 on IL-2R-expressing lung endothelial cells or CD4^+^ regulatory T cells^42^. On this note, ablation of the PGE_2_–EP_2_/EP_4_ signalling axis to enhance IL-2 responsiveness in adoptively transferred cancer-specific CD8^+^ T cells bears the promise to unleash their full potential to mount protective anticancer immunity not only in mice but also in cancer patient-derived TILs, as demonstrated in the accompanying paper^37^. Given the association of increased COX-mediated PGE_2_-production in tumours with cancer growth and poor survival rates in patients with cancer, our findings therefore identify the PGE_2_–EP_2_/EP_4_ signalling axis in TILs as molecular target to improve T cell immune therapy in cancer patients with PGE_2_-producing tumours. This strategy may further be beneficial in tumours that produce high levels of other EP_2_/EP_4_-engaging prostanoids such as PGF_2α_, PGD_2_ and PGI_2_ (ref. ^43^).

## Methods

### Mice

All mice used in this study were on a C57BL/6J genetic background and purchased from the Jackson Laboratory (JAX). OT-I × CD45.1 mice were generated by crossing OT-I mice (JAX, 003831) to CD45.1 (JAX, 002014) mice. *Ptger2*^*−/−*^*Ptger4*^*fl/fl*^ mice were generated by crossing *Ptger2*^*−/−*^ mice (JAX, 004376) to *Ptger4*^*fl/fl*^ mice (JAX, 028102) and further crossed to *Cd4*^*cre*^ mice (JAX, 022071) to generate *Cd4*^*cre*^*Ptger2*^*−/−*^*Ptger4*^*fl/fl*^ mice or crossed to *Gzmb*^*cre*^ mice (JAX, 003734) to generate *Gzmb*^*cre*^*Ptger2*^*−/−*^*Ptger4*^*fl/fl*^ mice. Unless stated otherwise, mice were on a CD45.2/CD45.2 background. For some experiments, *Cd4*^*cre*^*Ptger2*^*−/−*^*Ptger4*^*fl/fl*^ mice and *Ptger2*^*−/−*^*Ptger4*^*fl/fl*^ mice were crossed to OT-I mice to generate *Cd4*^*cre*^*Ptger2*^*−/−*^*Ptger4*^*fl/fl*^ OT-I mice and *Ptger2*^*−/−*^*Ptger4*^*fl/fl*^ OT-I mice and used on a CD45.1/CD45.2 or CD45.1/CD45.1 background. WT or *Rag1*^*−/−*^ mice (JAX, 002216) on a CD45.2/CD45.2 background were used as recipients in adoptive transfer experiments. In all experiments, mice at 6–12 weeks of age were sex-matched and randomly assigned to control or treatment groups. Mouse experiments with *Ptgs1/Ptgs2*^*−/−*^ BRAF^V600E^ tumours and T cell depletion were conducted without blinding; all other experiments were performed in a blinded manner. No statistical methods were used to predetermine sample sizes. Mice were killed by cervical dislocation under anaesthesia. All mice were maintained and bred at the Klinikum rechts der Isar, TUM, or at the Klinikum der Universität München, LMU, under specific-pathogen-free, controlled conditions with a 12-h light–dark cycle, ambient temperature of 24 °C and humidity maintained at 55%, and in accordance with the guidelines of the Federation of European Laboratory Animal Science Associations. All animal experiments were performed in accordance with the guidelines of the district government of upper Bavaria (Department 5–Environment, Health and Consumer Protection).

### Cell lines

Control and *Ptgs1/Ptgs2*^*−/−*^ BRAF^V600E^ melanoma cells were generated using the CRISPR–Cas9 system as previously described^14^. BRAF^V600E^-OVA and *Ptgs1/Ptgs2*^*−/−*^ BRAF^V600E^-OVA cells were generated by lentiviral transduction. In brief, OVA cDNA was subcloned into a pHIV-7 transfer vector carrying both the phosphoglycerate kinase (*PGK*) promoter and IRES-puromycin-resistance sequence. The production of third-generation self-inactivating lentiviral vectors, pseudotyped with VSV.G, was carried out as previously described^44^. Specifically, packaging cells were transfected and, after 2 days, cell supernatants were collected, filtered and used to transduce tumour cell lines in the presence of 8 µg ml^–1^ polybrene (Merck). After the incubation period, medium was exchanged for fresh medium, and target cells were passaged at least three times after transduction and selected using puromycin. MC38 cells were provided by A. Krüger, Institute of Experimental Oncology, TUM, and MC38-OVA and Panc02 cells were provided by V. Buchholz, Institute for Medical Microbiology, Immunology and Hygiene, TUM.

BRAF^V600E^, *Ptgs1/Ptgs2*^*−/−*^ BRAF^V600E^, BRAF^V600E^-OVA and *Ptgs1/Ptgs2*^*−/−*^ BRAF^V600E^-OVA cells were cultured in complete RPMI medium (RPMI 1640 medium (Thermo Fisher Scientific) supplemented with 10% FCS (Merck), 50 µM β-mercaptoethanol (Thermo Fisher Scientific), 50 U ml^–1^ penicillin (Thermo Fisher Scientific), 50 µg ml^–1^ streptomycin (Thermo Fisher Scientific) and 2 mM l-glutamine (Thermo Fisher Scientific). D4M.3A-pOVA cells were generated as previously described^45^ and cultured in DMEM-F12 medium (Thermo Fisher Scientific). MC38, MC38-OVA and Panc02 cells were cultured in DMEM (Thermo Fisher Scientific), with both media supplemented with 10% FCS, 50 µM β-mercaptoethanol, 50 U ml^–1^ penicillin, 50 mg ml^–1^ streptomycin, 2 mM l-glutamine, 1× MEM non-essential amino acids solution (Thermo Fisher Scientific) and 1 mM sodium pyruvate (Thermo Fisher Scientific). To generate tumour cell conditioned medium (CM), 5 × 10^6^ tumour cells were cultured in 20 ml complete RPMI medium for 48 h and the supernatant was collected, filtered and stored at −20 °C until further use. All cell lines were routinely tested for mycoplasma contamination in-house by PCR. For *Ptgs1/Ptgs2*^*−/−*^ BRAF^V600E^ cells, the absence of PGE_2_ production was routinely confirmed by PGE_2_ ELISA (Cayman Chemical). No further cell line authentications were conducted in the laboratory.

### Tumour cell injections

Tumour cell lines were detached by trypsinization (Thermo Fisher Scientific) and washed three times in sterile PBS (Thermo Fisher Scientific). Unless stated otherwise, 2 × 10^6^ cells were injected s.c. in 100 µl sterile PBS into the flank of each recipient mouse. Tumour growth was measured using a digital calliper. Tumour diameters stated in the figures refer to the average values of the longest diameter and its perpendicular for each tumour. A maximal tumour diameter of 15 mm served as the humane end point and was not exceeded in any of the experiments.

### CD4^+^ and CD8^+^ T cell depletion in vivo

To deplete CD4^+^ and CD8^+^ T cells, mice received intraperitoneal (i.p.) injections of 100 µl anti-mouse CD4 (100 µg per mouse, GK1.5, BioXCell) or anti-mouse CD8β (100 µg per mouse, 53-5.8, BioXCell) antibodies every 5 days, beginning on day 1 following tumour cell inoculation.

### FTY720 treatment in vivo

FTY720 treatment was performed by injecting mice i.p. with 100 µl of FTY720 (20 µg per mouse, Merck) on day 1 or day 6 after tumour cell transplantation. Injection with 100 µl sterile isotonic NaCl served as control.

### IL-2 receptor blockade in vivo

For blockade of IL-2Rβ and IL-2Rγc, mice received i.p. injections of 150 µl anti-mouse CD122 (300 µg per mouse, TM-Beta 1, BioXCell) and anti-mouse CD132 (300 µg per mouse, 3E12, BioXCell) antibodies on days 6 and 7 after tumour cell transplantation. Injections with 150 µl sterile isotonic NaCl served as control.

### Processing of tumour tissue and lymphoid organs

Tumours, tdLNs or spleens of tumour-bearing mice were excised at the indicated time points after cell transplantation. Tumour or organ weight was determined using a microscale. For subsequent analyses by flow cytometry or cell sorting, tumour samples were mechanically dissociated and incubated with collagenase IV (200 U ml^–1^, Thermo Fisher Scientific) and DNase I (100 µg ml^–1^, Merck) for 40 min at 37 °C and filtered through a 70 µm and a 30 µm cell strainer (Miltenyi) to generate single-cell suspensions. Spleens were passed through a 70 µm cell strainer, followed by red blood cell lysis and a second filtration step using a 30 µm cell strainer. LNs were passed through a 30 µm cell strainer. For the isolation of migratory cDC1s, LNs were processed as described for tumour samples.

### Antibodies and reagents for flow cytometry and cell sorting

The following antibodies and staining reagents were used for flow cytometry or cell sorting: fixable viability dye eFluor 450 (dilution: 1:500; Thermo Fisher Scientific); fixable viability dye APC-eF780 (1:1,000; Thermo Fisher Scientific); viability dye SYTOX-blue (1:2,000; Thermo Fisher Scientific); APC anti-CD3 (1:100; clone 17A2, Thermo Fisher Scientific); PE anti-CD4 (1:200; GK1.5, Biolegend); AF647 anti-CD4 (1:200; GK1.5, Biolegend); PerCP/Cy5.5 anti-CD4 (1:200; GK1.5, Biolegend); BV421 anti-CD8α (1:200; 53-6.7, Biolegend); FITC anti-CD8α (1:200; 53-6.7, Biolegend); PE-Dazzle594 anti-CD8α (1:200; 53-6.7, Biolegend); PE-Cy7 anti-CD8α (1:200; 53-6.7, Biolegend); BV605 anti-CD11b (1:200; M1/70, Biolegend); PE-Cy7 anti-CD11c (1:200; N418, Biolegend); BV570 anti-mouse/human-CD44 (1:100; IM7, Biolegend); BV711 anti-mouse/human-CD44 (1:100; IM7, Biolegend); FITC anti-mouse/human-CD44 (1:100; IM7, Biolegend); PerCP-Cy5.5 anti-mouse/human-CD44 (1:100; IM7, Biolegend); AF647 anti-CD45.1 (1:100; A20, Biolegend); PE anti-CD45.1 (1:100; A20, Biolegend); PE-Dazzle594 anti-CD45.1 (1:100; A20, Biolegend); PerCP/Cy5.5 anti-CD45.1 (1:100; A20, Biolegend); BV510 anti-CD45.2 (1:100; 104, Biolegend); FITC anti-CD45.2 (1:100; 104, Biolegend); PerCP-Cy5.5 anti-CD45.2 (1:100; 104, Biolegend); FITC anti-CD62L (1:100; MEL-14, Biolegend); PE-Dazzle594 anti-CD62L (1:100; MEL-14, Biolegend); FITC anti-CD103 (1:100; M290, BD Biosciences); APC anti-CD132/IL2Rγc (1:100; TUGm2, Biolegend); PE-Dazzle594 anti-CD186/CXCR6 (1:200; SA051D1, Biolegend); PE anti-CX_3_CR1 (1:100; SA011F11, Biolegend); BV605 anti-CD279/PD-1 (1:100; 29 F.1A12, Biolegend); BV421 anti-CD366/TIM-3 (1:200; RMT3-23, Biolegend); PerCP/Cy5.5 anti-TCRβ (1:100; H57-597, Biolegend); AF700 anti-I-A/I-E (1:500; MHC class II) (M5/114.15.2, Biolegend); PE anti-H-2K^b^ bound to SIINFEKL (1:100; 25-D1.16, Biolegend); APC anti-human GZMB (1:200; GB12, Thermo Fisher Scientific); FITC anti-Ki-67 (1:100; SolA-15, Thermo Fisher Scientific); AF700 anti-Ki-67 (1:100; SolA-15, Thermo Fisher Scientific); PE anti-TCF1/TCF7 (1:40; S33-966, BD Biosciences); AF488 anti-human pSTAT5 (0.03 µg per test, 47/Stat5(pY694); BD Biosciences); eF660 anti-TOX (1:100; TXRX10, Thermo Fisher Scientific); eFluor660 Rat-IgG2a-κ isotype-control (1:100; eBR2a, Thermo Fisher Scientific); APC mouse-IgG1κ isotype-control (1:200; P3.6.2.8.1, Thermo Fisher Scientific); AF488 mouse-IgG1κ isotype-control (0.03 µg per test; MOPC-21, Biolegend); and rabbit-anti-mouse-TCF1/TCF7 (1:100; C.725.7, Thermo Fisher Scientific). These were followed by AF647 donkey-anti-rabbit IgG (1:200; Poly4064, Biolegend) or DL488 donkey-anti-rabbit IgG (1:200; Poly4064, Biolegend). Unless stated otherwise, all antibodies were anti-mouse antibodies.

### Flow cytometry and cell sorting

For staining of surface markers and viability dyes, cells were stained for 15 min at 4 °C in FACS buffer (PBS with 1% FCS and 2 mM EDTA). Staining of SIINFEKL–MHC class I complexes on cDC1s for analysis of OVA cross-presentation was performed for 40 min. For intracellular staining of GZMB, TCF1, Ki-67 and TOX, cells were fixed and permeabilized using the True-Nuclear Transcription Factor Buffer Set (Biolegend) according to the manufacturer’s protocol. Intracellular staining was performed overnight in permeabilization buffer at 4 °C. For intracellular staining of pSTAT5, cells were fixed and permeabilized using BD Cytofix (BD Biosciences) and BD Phosflow Perm Buffer III (BD Biosciences) according to the manufacturer’s instructions (protocols II and III, BD Biosciences). For the detection of EdU incorporation, EdU was added to the culture at a final concentration of 15 µM for the last 3 h of the experiment, and analysis was performed using an EdU Proliferation kit (iFluor 488, Abcam) according to the manufacturer’s protocol.

Flow cytometry analyses were performed using a LSR Fortessa Cell Analyzer (BD Biosciences, BD FACSDiva software v.8.0.1 and v.9.0.1), a SP6800 Spectral Cell Analyzer (Sony Biotechnologies, spectral analyser software v.2.0.2.14140) or a SA3800 Spectral Cell Analyzer (Sony Biotechnologies, spectral analyser software v.2.0.5.54250). For flow cytometric quantification of cell numbers, CountBright Absolute Counting Beads (Thermo Fisher Scientific) were added to samples before analyses. For some experiments, CD8^+^ TILs (live CD45^+^CD3^+^CD8^+^ cells), stem-like *Cd4*^*cre*^*Ptger2*^*−/−*^*Ptger4*^*fl/fl*^ OT-I TILs (live CD45.1^+^CD8^+^CD44^+^TIM-3^*−*^CXCR6^*−*^) or differentiated effector *Cd4*^*cre*^*Ptger2*^*−/−*^*Ptger4*^*fl/fl*^ OT-I TILs (live CD45.1^+^CD8^+^CD44^+^TIM-3^+^CXCR6^+^) were sorted using a FACS Aria III Cell Sorter (BD Biosciences, BD FACSDiva software v.9.0.1). Naive OT-I T cells (CD45.1^+^CD8^+^CD62L^+^CD44^–^) used in adoptive transfer experiments were sorted from blood using a SH800S Cell Sorter (Sony Biotechnologies, cell sorter software v.2.1.6). All flow cytometric data were analysed using FlowJo (BD Biosciences, v.00.8.1 and v.10.8.2).

### Adoptive T cell transfer

For adoptive T cell transfer of naive T cells, 1 × 10^3^ congenically marked naive CD8^+^ T cells from OT-I, *Ptger2*^*−/−*^*Ptger4*^*fl/fl*^ OT-I or *Cd4*^*cre*^*Ptger2*^*−/−*^*Ptger4*^*fl/fl*^ OT-I donor mice were injected i.v. in sterile PBS into sex-matched recipient WT mice 6 h before tumour cell transplantation s.c. For adoptive transfer of CRISPR-Cas9-edited T cells, 1 × 10^3^ cells congenically marked OT-I T cells from in vitro T cell cultures were injected i.v. into recipient mice at day 2 after tumour cell transplantation s.c. For re-transfer of CD8^+^ TILs, 7 × 10^3^ congenically marked stem-like (TIM-3^–^CXCR6^–^) or differentiated effector (TIM-3^+^CXCR6^+^) *Cd4*^*cre*^*Ptger2*^*−/−*^*Ptger4*^*fl/fl*^ OT-I TILs were sorted from MC38-OVA tumours from WT mice and injected i.v. in sterile PBS into sex-matched recipient *Rag1*^*−/−*^ mice inoculated with MC38-OVA tumour cells 2 days before T cell re-transfer.

### Generation of repetitively activated antigen-experienced TCF1^+^CD8^+^ T cells

TCF1^+^CD8^+^ T cells were differentiated from splenic naive CD8^+^ T cells by repetitive activation as previously described^35^, with minor modifications. In brief, 1 × 10^6^ naive CD8^+^ T cells were seeded in complete RPMI medium supplemented with 1× MEM non-essential amino acids solution and 1 mM sodium pyruvate. Low-dose IL-2 (85 U ml^–1^) and mouse anti-CD3/CD28 microbeads were added to the culture while maintaining a CD8^+^ T cell concentration of 1 × 10^6^ cells per ml for multiple (re-)activation cycles over a course of 4 days, followed by purification of live cells by gradient centrifugation (Pancoll).

### T cell effector differentiation

Effector differentiation of TCF1^+^CD8^+^ T cells was performed as previously described^35^, with minor modifications. In brief, cells were cultured with mouse anti-CD3/CD28 microbeads in the presence of high-dose IL-2 (350 U ml^–1^). Where indicated, PGE_2_ (100 ng ml^–1^, unless indicated otherwise in the figure legend; Thermo Fisher Scientific), tumour cell CM, IL-7 (10 ng ml^–1^, Miltenyi), IL-12 (10 ng ml^–1^, Biolegend) or IL-15/15Rα (1 ng ml^–1^, Thermo Fisher Scientific) was added to the culture. To assess T cell expansion, the numbers of live CD45^+^CD3^+^CD8^+^ T cells were quantified by flow cytometry 72 h after the incubation period.

### Gene deletion by CRISPR–Cas9–gRNA complex electroporation

*Cd4*^*cre*^*Ptger2*^*−/−*^*Ptger4*^*fl/fl*^ OT-I T cells were purified from spleen and cultured in complete RPMI supplemented with IL-2 (10 U ml^–1^) and IL-7 (5 ng ml^–1^) in the presence of mouse anti-CD3/CD28 microbeads. After 24 h, anti-CD3/CD28 microbeads were removed by magnetic separation and cells were electroporated (4D-Nucleofector, Lonza; pulse program CM137)^46^ in P3 electroporation buffer supplemented with the Cas9 electroporation enhancer (IDT), Cas9 protein (IDT) and *Cd122*-targeting or non-targeting gRNAs. gRNAs were generated by hybridizing trRNA (IDT) with *Cd122*-targeting (sequences TATGTCAAGGAGGTCCACGG and CTGGGAACGACCCGAGGATC, generated using CHOPCHOP; ref. ^47^) or non-targeting crRNA (IDT) (GCCTGCCCTAAACCCCGGAA; ref. ^48^) as mock control. Cells were rested in complete RPMI supplemented with IL-7 (5 ng ml^–1^, Miltenyi) at 37 °C for 48 h and validated for specific knockout by CD122 surface staining before injection into recipient mice.

### Analysis of IL-2Rγc expression and IL-2 signalling

TCF1^+^CD8^+^ T cells from in vitro cultures were rested for 20 h in complete RPMI supplemented with low-dose IL-2 and purified by gradient centrifugation. Cells were stimulated with mouse anti-CD3/CD28 microbeads and low-dose IL-2 for 24 h in the absence or presence of PGE_2_ (100 ng ml^–1^). After 24 h, IL-2Rγc chain expression was analysed by flow cytometry. For analysis of IL-2-induced STAT5 signalling, anti-CD3/CD28 microbeads were removed by magnetic separation, cells were rested for 30 min at 37 °C in complete RPMI and stimulated for 30 min with different concentrations of IL-2 (10–100 U ml^–1^, as indicated). After the incubation period, fixation buffer was directly added to the culture to terminate the signalling process and cells were stained for flow cytometry analysis.

### PGE_2_ measurements

Tumours and organs of tumour-bearing mice were excised 11 days after tumour cell transplantation, directly frozen in liquid nitrogen and stored at −80 °C until further processing. Samples were homogenized in homogenization buffer (0.1 M PBS, 1 mM EDTA and 10 µM indomethacin (Merck), pH 7.4) using a gentleMACS Dissociator (Miltenyi) followed by a freeze–thaw cycle. PGE_2_ concentrations were measured by ELISA (Cayman Chemical) according to the manufacturer’s protocol.

### RNA isolation and quantitative real-time PCR

RNA was isolated using an Arcturus PicoPure RNA isolation kit (Thermo Fisher Scientific) and cDNA was generated using a SensiFAST cDNA synthesis kit (Bioline). Quantitative real-time PCR was carried out on a LightCycler 480 (Roche, LightCycler 480 software v.1.5.1) using a TAKYON No ROX SYBR MasterMix dTTP Blue kit (Eurogentec) according to the manufacturer’s protocol. *Ptger4* expression was determined using the ΔCt method, with *Hprt* serving as reference gene. Primer sequences were from a previous study^38^. All primers were purchased from Eurofins.

### scRNA-seq and scTCR-seq

CD8^+^ TILs were sorted from BRAF^V600E^ tumours 11 days after tumour cell transplantation. A combination of cell hashing and DNA barcoding during library preparation was used for sample multiplexing, which enabled the simultaneous sequencing of four biological replicates from each group. For cell hashing, unique TotalSeq-C anti-mouse hashtag antibodies were used for hashing of cells from each experimental group as follows: WT: hashtag 1; *Ptger2*^*−/−*^*Ptger4*^*fl/fl*^: hashtag 2; *Cd4*^*cre*^*Ptger2*^*−/−*^*Ptger4*^*fl/fl*^: hashtag 3; and *Gzmb*^*cre*^*Ptger2*^*−/−*^*Ptger4*^*fl/fl*^: hashtag 4 (1:250 each, Biolegend). Hashtagged cells from one tumour-bearing mouse of each group were pooled and loaded on a Chromium Next GEM Chip (10x Genomics). RNA-seq libraries were generated using Chromium Next GEM Single Cell 5′ Reagent kits v.2 User Guide with Feature Barcode technology for Cell Surface Protein (Rev D) according to the manufacturer’s protocol (10x Genomics). Quality control was carried out using a High Sensitivity DNA kit (Agilent), a Bioanalyzer 2100 and a Qubit dsDNA HS Assay kit (Thermo Fisher Scientific). For sequencing, libraries were pooled and analysed by paired-end sequencing (2 × 150 bp) on a NovaSeq6000 platform using S4 v.1.5 (300 cycles) sequencing kits (Illumina). Libraries were sequenced to a depth of at least 2 × 10^4^ reads per cell for gene expression libraries and 5 × 10^3^ reads per cell for T cell receptor libraries.

Initial scRNA-seq analyses were performed for all samples from the groups *Ptger2*^*−/−*^*Ptger4*^*fl/fl*^, *Cd4*^*cre*^*Ptger2*^*−/−*^*Ptger4*^*fl/fl*^ and *Gzmb*^*cre*^*Ptger2*^*−/−*^*Ptger4*^*fl/fl*^, with data from the WT group being added at a later stage for validation of *Ptger2* and *Ptger4* read coverage (see below). Alignment of gene expression libraries and demultiplexing were performed using cellranger multi (Cell Ranger (v.6.1.1)^49^; 10x Genomics) against the pre-built mouse reference v2020-A (10x Genomics, mm10/GRCm38, annotation from GENCODE Release M23) with the number of expected cells equals 21.000 as input argument. The BAM files were converted to FASTQ files using the tool bamtofastq with the argument --reads-per-fastq set to the total number of reads in the BAM file plus 10,000. After that, gene expression and TCR analysis were combined by running cellranger multi separately for each demultiplexed sample, disabling library concordance reinforcement. The algorithm was forced to find the number of cells identified in the first step of demultiplexing, and sample-specific FASTQ files were used as input for the gene expression analysis pipeline. The pre-built Ensembl GRCm38 Mouse V(D)J Reference v.5.0.0 was used for TCR analysis.

The initial downstream analysis was performed in R (v.4.0.4) with the R package Seurat (v.4.0.1)^50^. Only cells with more than 1,000 genes detected, less than 10% of mitochondrial genes and with UMI counts less than 3 standard deviations above the mean were kept. The data were filtered for genes detected in at least three cells in one of the samples. Filtered read counts from each sample were normalized independently using sctransform (v.0.3.2)^51^ with the glmGamPoi method^52^. Anchors between cells from different replicates were identified on the top 1,000 highly variable genes using canonical correlation analysis and 30 canonical vectors. Data integration was performed on first 20 PC analysis (PCA) dimensions. PCA was calculated for the integrated data on the top 1,000 highly variable genes and both *k*-nearest neighbour graph and UMAP were computed on the 30 nearest neighbours and first 20 PCA dimensions. Louvain clusters were identified using the shared nearest neighbour modularity optimization-based algorithm at resolutions 0.9, 0.65 and 0.9 for the groups *Ptger2*^*−/−*^*Ptger4*^*fl/fl*^, *Cd4*^*cre*^*Ptger2*^*−/−*^*Ptger4*^*fl/fl*^ and *Gzmb*^*cre*^*Ptger2*^*−/−*^*Ptger4*^*fl/fl*^, respectively. Contaminating myeloid cells were identified based on the average cluster expression of the marker genes *Cd14*, *Lyz2*, *Fcgr3*, *Ms4a7*, *Fcer1g*, *Cst3*, *H2-Aa*, *Ly6d*, *Ms4a1* and *Ly6d*. Cycling cells were identified based on expression of *Cdk1*, *Mcm2*, *Pclaf*, *H2afz*, *Birc5* and *Mki67*.

The integrative analysis between groups was performed in R (v.4.2.1) with the R package Seurat (v.4.1.1)^50^. After general data pre-processing and regression of contaminating cells as mentioned above, filtered read counts from each sample were normalized independently using sctransform (v.0.3.2)^51^ with glmGamPoi method^52^. Anchors between cells from all groups and all their replicates were identified using a more conservative approach, which led to weaker batch correction. For that purpose, reciprocal PCA was applied on the top 1,000 highly variable genes of each sample and anchors were picked using the first 20 dimensions and 1 neighbour only. PCA was performed on the integrated data on the top 1,000 highly variable genes. A *k*-nearest neighbour graph and UMAP (spread of 0.4, minimum distance of 0.01) were computed on the first 20 PCs and 30 nearest neighbours. A resolution of 0.6 was used for Louvain clusters identification using the shared nearest neighbour modularity optimization-based algorithm. DEGs between two groups were identified using the Wilcoxon rank-sum test and Bonferroni correction. Gene set expression scores at single-cell level were calculated using the AddModuleScore function, including only the detected genes. Similarity scores with reference datasets were calculated using the R package SingleR (v.1.10.0)^53^ with the top 200 DEGs. The processed transcriptome profiles of naive CD8^+^ T cells, memory stem cell CD8^+^ T cells and central memory CD8^+^ T cells were from a previous study^54^. For tumour antigen-specific CD8^+^ T cells in tdLNs, tumour-infiltrating stem-like CD8^+^ T cells and their naive counterparts, data from a previous study^3^ were processed using the R package DESeq2 (v.1.36)^55^. Gene set expression scores at the single-cell level were calculated using the AddModuleScore function, including only the detected genes. The effector T cell gene signature was from a previous study^56^ (M3013: KAECH_NAIVE_VS_DAY8_EFF_CD8_TCELL_DN). The CD8^+^ T cell proliferation signature was obtained from MSigDB (GO:2000566). Transcriptional trajectories were inferred using the R package slingshot (v.2.4.0)^57^ over the UMAP calculated on the integrated data, approximating the curves by 150 points. The pseudotime was calculated as a weighted average across lineages, weighted by the assignment weight.

TCR analysis of clonotype was performed using the R package scRepertoire (v.1.6.0)^58^. Clonotypes were called based on a combination of VDJC genes comprising the TCR and the nucleotide sequence of the CDR3 region. Whenever the clonotype distribution is shown for individual groups, the cell number was downsampled, so that cluster 1 from all groups had the same maximum size. TF activity was inferred using the weighted mean method of decoupleR (v.2.2.2)^59^ and TF–target interactions available through dorothea (v.1.8.0)^60^, with confidence levels A to C. Normalization to *Ptger2*^*−/−*^*Ptger4*^*fl/fl*^ was achieved by subtracting its scores from the scores of the other groups. The top 100 variable TFs between clusters within each group were used to draw a network graph with tidygraph (v.1.2.1)^61^ based on common targets with same defined mode of regulation as defined on the database. Only TFs with at least two common targets were kept for visualization. Louvain clusters were identified using igraph (v.1.3.2)^62^ at a resolution of 0.5.

For addition of scRNA-seq data from the WT group, samples were pre-processed as described above and mapped to a reference formed by the integrated data of the *Ptger2*^*−/−*^*Ptger4*^*fl/fl*^, *Cd4*^*cre*^*Ptger2*^*−/−*^*Ptger4*^*fl/fl*^ and *Gzmb*^*cre*^*Ptger2*^*−/−*^*Ptger4*^*fl/fl*^ groups using the R package Seurat (v.4.1.1)^50^. For that purpose, anchors between cells from the reference and the WT groups along with all replicates were identified using reciprocal PCA on top 1,000 highly variable genes. Anchors were picked using the first 20 dimensions and 1 neighbour only. Annotations were transferred using the function TransferData, and data were integrated using IntegrateEmbeddings. Cells from the added group were then projected onto the coordinates of the reference UMAP calling ProjectUMAP with 30 nearest neighbours. Read coverage was estimated using deepTools (v.3.5.4)^63^ with bamCoverage and a bin size of 10 bp and normalization by bins per million mapped reads. For coverage analysis on *Tcf7*/TCF1^+^ and *Tcf7*/TCF1^*−*^ clusters, BAM files were split by cell barcodes from clusters 1–2 or clusters 3–8 using samtools (v.1.13)^64^ before coverage estimation. Read coverage on gene tracks was visualized using the R package trackViewer (v.1.32.1)^65^.

### RNA-seq

In vitro generated, repetitively activated TCF1^+^CD8^+^ T cells were incubated in the presence or absence of PGE_2_ (100 ng ml^–1^) for 1 h at 37 °C followed by stimulation with IL-2 or IL-2 plus mouse anti-CD3/CD28 microbeads for an additional 4 h. Total RNA was isolated using Total RNA Miniprep (Monarch). Library preparation was carried out using a NEB Next UltraRNA Library Prep kit with i7 and i5 index reads of 8 bp each for mRNA library preparation and poly A enrichment. Sequencing was performed on a NovaSeq6000 PE150 platform in paired-end mode (read 1: 151 bp, read 2: 151 bp), using S4 (v.1.5) (300 cycles) sequencing kits (Illumina). Reads were aligned to the mouse reference genome (GRCm38/mm10, NCBI) using the Hisat2 (v.2.0.5) mapping tool. To quantify gene expression levels, featureCounts (v.1.5.0-p3) was used to count the reads mapped to each gene, followed by the calculation of fragments per kilobase of transcript sequence per million mapped reads based on gene length and read count. DEGs were identified using the DESeq2 R package (v.1.20.0). Adjusted *P* values were obtained using Wald test with multiple testing by the Benjamini–Hochberg method, and genes identified by DESeq2 with adjusted *P* values < 0.05 and fold change ≥ 2 were assigned as DEGs. Volcano plots were visualized using the ggplot2 R package ggplot2 (v.3.4.2), and PCA was conducted using the prcomp function in R and visualized using the R packages ggplot2 and ggrepel (v.0.9.3). DEGs obtained from comparing the groups ‘anti-CD3/CD28 +IL-2’ and ‘PGE_2_-treated + anti-CD3/CD28 +IL-2’ were ordered based on their log_2_ fold change values and subjected to GSEA using GSEA (v.4.3.2) probing for hallmark genes from mh.all.v2023.1.Mm (MSigDB). The PreRanked tool from GSEA (v.4.3.2) was used to determine the NES and significance by adjusted *P* values.

### Statistical analyses

The GraphPad Prism software (v.9.5.0 and v.9.5.1) was used for statistical analyses. Affinity Designer (v.1.10.6) (Serif) was used to visualize data. Paired or unpaired two-tailed Student’s *t*-test, one-way ANOVA or two-way ANOVA was used to assess statistical significance, as indicated in in the figure legends. Data are shown as the mean ± s.d., mean ± s.e.m. or box and whiskers plots, as indicated in the figure legends.

### Reporting summary

Further information on research design is available in the Nature Portfolio Reporting Summary linked to this article.

## Online content

Any methods, additional references, Nature Portfolio reporting summaries, source data, extended data, supplementary information, acknowledgements, peer review information; details of author contributions and competing interests; and statements of data and code availability are available at 10.1038/s41586-024-07254-x.

### Supplementary information

Reporting Summary

### Source data

Source Data Fig. 1

Source Data Fig. 2

Source Data Fig. 3

Source Data Fig. 4

Source Data Extended Data Fig. 1

Source Data Extended Data Fig. 2

Source Data Extended Data Fig. 3

Source Data Extended Data Fig. 5

Source Data Extended Data Fig. 6

Source Data Extended Data Fig. 7

Source Data Extended Data Fig. 8

Source Data Extended Data Fig. 9

## Data Availability

Data from scRNA-seq and scTCR-seq of CD8^+^ TILs and data from RNA-seq of TCF1^+^CD8^+^ T cells from in vitro T cell cultures have been deposited into the Gene Expression Omnibus (GEO; https://www.ncbi.nlm.nih.gov/gds) under the superseries number GSE231340 (subseries numbers GSE231301 and GSE231302). The pre-built mouse reference v2020-A was provided by 10x Genomics (downloaded from https://cf.10xgenomics.com/supp/cell-exp/refdata-gex-GRCh38-2020-A.tar.gz) and is based on the mm10 GRCm38.p6 release 98 from Ensembl (http://ftp.ensembl.org/pub/release-98/fasta/mus_musculus/dna/Mus_musculus.GRCm38.dna.primary_assembly.fa.gz) with reference annotation from GENCODE Release M23 (http://ftp.ebi.ac.uk/pub/databases/gencode/Gencode_mouse/release_M23/gencode.vM23.primary_assembly.annotation.gtf.gz) provided by 10x Genomics. The pre-built GRCm38 Mouse V(D)J Reference v.5.0.0 was provided by 10x Genomics and downloaded from https://cf.10xgenomics.com/supp/cell-vdj/refdata-cellranger-vdj-GRCm38-alts-ensembl-5.0.0.tar.gz. Source data are provided with this paper.
