# Supplementary material for: PGE2 limits effector expansion of tumour-infiltrating stem-like CD8+ T cells
Source: Nature. 2024 Apr 24;629(8011):417–25. doi: 10.1038/s41586-024-07254-x (PMC11078747; doi:10.1038/s41586-024-07254-x)
Supplement: Supplementary file 1 — Reporting Summary [file 41586_2024_7254_MOESM1_ESM.pdf]

nature portfolio

Corresponding author(s): Jan P. Böttcher

Last updated by author(s): Feb 19, 2024

Reporting Summary

Nature Portfolio wishes to improve the reproducibility of the work that we publish. This form provides structure for consistency and transparency in reporting. For further information on Nature Portfolio policies, see our Editorial Policies and the Editorial Policy Checklist.

Statistics

For all statistical analyses, confirm that the following items are present in the figure legend, table legend, main text, or Methods section.

n/a	Confirmed	
☐	☒	The exact sample size (*n*) for each experimental group/condition, given as a discrete number and unit of measurement
☐	☒	A statement on whether measurements were taken from distinct samples or whether the same sample was measured repeatedly
☐	☒	The statistical test(s) used AND whether they are one- or two-sided<br>*Only common tests should be described solely by name; describe more complex techniques in the Methods section.*
☐	☒	A description of all covariates tested
☐	☒	A description of any assumptions or corrections, such as tests of normality and adjustment for multiple comparisons
☐	☒	A full description of the statistical parameters including central tendency (e.g. means) or other basic estimates (e.g. regression coefficient) AND variation (e.g. standard deviation) or associated estimates of uncertainty (e.g. confidence intervals)
☐	☒	For null hypothesis testing, the test statistic (e.g. *F*, *t*, *r*) with confidence intervals, effect sizes, degrees of freedom and *P* value noted<br>*Give P values as exact values whenever suitable.*
☐	☒	For Bayesian analysis, information on the choice of priors and Markov chain Monte Carlo settings
☐	☒	For hierarchical and complex designs, identification of the appropriate level for tests and full reporting of outcomes
☐	☒	Estimates of effect sizes (e.g. Cohen's *d*, Pearson's *r*), indicating how they were calculated

Our web collection on statistics for biologists contains articles on many of the points above.

Software and code

Policy information about availability of computer code

Data collection	Flow cytometer data was collected on a LSRFortessa Cell Analyzer using BD FACSDiva Software v8.0.1 and v9.0.1 (BD Biosciences), a SP6800 using SP6800 Spectral Analyzer Software v2.0.2.14140 (SONY Biotechnology) or a SA3800 Spectral Analyzer using SA3800 Spectral Analyzer Software v2.0.5.54250 (SONY Biotechnology). Fluorescence activated cell sorting of T cells was performed on a SH800 Cell Sorter using SH800 Cell Sorter v2.1.6 (SONY Biotechnology) or a BD FACSAria III Cell Sorter using BD FACSDiva v9.0.1.<br><br>qRT-PCR was performed on a LightCycler 48 (Roche) using LightCycler 480 Software v1.51.<br><br>PGE2 concentrations were measured on a Berthold Tristar 3 using MikroWin v5.24.<br><br>Bulk RNA was isolated using Total RNA Miniprep (Monarch).<br>Bulk RNA sequencing was performed using the NovaSeq6000 (Illumina).<br><br>Single-cell RNA was isolated using the Chromium Next GEM Single Cell 5' Reagent Kits v2 User Guide with Feature Barcode technology for Cell Surface Protein (Rev D).<br>Single-cell RNA sequencing was performed using the NovaSeq6000 (Illumina).
Data analysis	The following softwares were used for data analysis:<br><br>Flow cytometry data: FACSDiva v8.0.1 and v9.0.1 (BD Biosciences), Flowjo v00.8.1 and v10.8.2 (BD Biosciences), SH800 Cell Sorter Software (SONY Biotechnology), SP6800 Spectral Cell Analyzer Software v2.0.2.14140 (SONY Biotechnology)

Data processing, visualisation and statistical analyses: Prism v9.5.0 and v9.5.1 (GraphPad), Excel v16.82 (Microsoft), Affinity Designer v1.10.6 (Serif)

RNA-seq: Reads were aligned using the Hisat2 v2.0.5, R v4.0.4 with the R packages: DESeq2 v1.20.0 and v1.36, featureCounts v1.5.0-p3, ggplot2 v3.4.2, ggrepel 0.9.3; GSEA: The PreRanked tool from GSEA v4.3.2 was used.

scRNA-seq and scTCR-seq: Cell Ranger v6.1.1, R v4.0.4 and R v4.2.1 with the R packages: Seurat v4.0.1 and v4.1.1, sctransform v0.3.2, slingshot v2.4.0, singleR v1.10.0, scRepertoire v1.6.0, decoupleR v2.2.2, dorothea v1.8.0, tidygraph v1.2.1, deepTools v3.5.4, samtools v1.13, trackViewer v1.32.1, and igraph v1.3.2. For the scRNA-seq: NovaSeq600 platform (S4 v1.5, Illumina).

For further details please see methods section.

For manuscripts utilizing custom algorithms or software that are central to the research but not yet described in published literature, software must be made available to editors and reviewers. We strongly encourage code deposition in a community repository (e.g. GitHub). See the Nature Portfolio guidelines for submitting code & software for further information.

Data

Policy information about availability of data

All manuscripts must include a data availability statement. This statement should provide the following information, where applicable:

- Accession codes, unique identifiers, or web links for publicly available datasets
- A description of any restrictions on data availability
- For clinical datasets or third party data, please ensure that the statement adheres to our policy

Data from single-cell RNA sequencing and single-cell TCR sequencing of tumour-infiltrating CD8+ T cells and data from RNA sequencing of TCF1+CD8+ T cells from in vitro T cell cultures is deposited at GEO under the SuperSeries GSE231340. This SuperSeries is composed of the following SubSeries:
GSE231301: RNA sequencing of TCF1+CD8+ T cells from in vitro T cell cultures
GSE231302: Single-cell RNA-sequencing and single-cell TCR-sequencing of tumour-infiltrating CD8+ T cells derived from mouse BRAFV600E melanoma [scRNA-Seq + TCR 10x]

The pre-built mouse reference v2020-A was provided by 10X Genomics, downloaded from https://cf.10xgenomics.com/supp/cell-exp/refdata-gex-GRCh38-2020-A.tar.gz and is based on the mm10 GRCm38.p6 release 98 from Ensembl (http://ftp.ensembl.org/pub/release-98/fasta/mus_musculus/dna/Mus_musculus.GRCm38.dna.primary_assembly.fa.gz) with reference annotation from GENCODE Release M23 (http://ftp.ebi.ac.uk/pub/databases/gencode/Gencode_mouse/release_M23/gencode.vM23.primary_assembly.annotation.gtf.gz) provided by 10x Genomics). The pre-built GRCm38 Mouse V(D)J Reference v5.0.0 was provided by 10X Genomics and downloaded from https://cf.10xgenomics.com/supp/cell-vdj/refdata-cellranger-vdj-GRCm38-alts-ensembl-5.0.0.tar.gz. Mathematical code for scRNA-seq, scTCR-seq and RNA-seq data analysis are available from the corresponding author upon reasonable request.

Human research participants

Policy information about studies involving human research participants and Sex and Gender in Research.

Reporting on sex and gender	N/A
Population characteristics	N/A
Recruitment	N/A
Ethics oversight	N/A

Note that full information on the approval of the study protocol must also be provided in the manuscript.

Field-specific reporting

Please select the one below that is the best fit for your research. If you are not sure, read the appropriate sections before making your selection.

☒ Life sciences ☐ Behavioural & social sciences ☐ Ecological, evolutionary & environmental sciences

For a reference copy of the document with all sections, see nature.com/documents/nr-reporting-summary-flat.pdf

Life sciences study design

All studies must disclose on these points even when the disclosure is negative.

Sample size	In order to determine appropriate sample sizes, we performed pilot experiments and referred to previously published results using the same or similar experimental models (Böttcher et al., 2018; Zelenay et al., 2015). No statistical methods were used to predetermine sample size. The exact n numbers for biological replicates used in the study are indicated in the respective figure legends.
Data exclusions	Every mouse designated for the respective experiment was included in the analysis. For flow cytometry, every tumour palpable at the analysis time point was analysed. Data on individual samples were excluded if any obvious problems during sample processing occurred, i.e. more

than 70% of dead cells identified by live dead staining. For scTCR-sequencing, sequencing for one replicate group failed and was therefore excluded from the analysis.

Replication

All animal experiments in this study were repeated and validated as stated in the respective figure legends.
All other experiments in this study, including ex vivo experiments, were repeated and validated as stated in the respective figure legends.
All attempts at replication were successful.

Randomization

Age- and sex-matched mice were randomly allocated into different groups and received the appropriate treatment at the same time point for comparative analyses. For experiments other than mice, every treatment condition included all samples, randomization was therefore not relevant.

Blinding

For mouse tumour experiments including Ptgs1/Ptgs2-/- BRAFV600E tumours as well as T cell depletion, investigators could not be blinded due to the overt differences in tumour size and weight, which reflected previously reported effects of these treatments in equal or similar experimental settings (Böttcher et al., Cell 2018). All other experiments were conducted in a blinded manner.
Every experiment was performed using several control samples and the applied analysis strategy (Immunofluorescence stainings, FACS gating) was identical for every single sample.

Reporting for specific materials, systems and methods

We require information from authors about some types of materials, experimental systems and methods used in many studies. Here, indicate whether each material, system or method listed is relevant to your study. If you are not sure if a list item applies to your research, read the appropriate section before selecting a response.

Materials & experimental systems

n/a	Involved in the study
☐	☒ Antibodies
☐	☒ Eukaryotic cell lines
☒	☐ Palaeontology and archaeology
☐	☒ Animals and other organisms
☒	☐ Clinical data
☒	☐ Dual use research of concern

Methods

n/a	Involved in the study
☒	☐ ChIP-seq
☐	☒ Flow cytometry
☒	☐ MRI-based neuroimaging

Antibodies

Antibodies used

The following antibodies were used for flow cytometry and cell sorting:
APC anti-CD3 (1:100, clone 17A2, Thermo Fisher Scientific, Cat.#: 17-0032-82), PE anti-CD4 (1:200, clone GK1.5, Biolegend, Cat.#: 100407), AF647 anti-CD4 (1:200, clone GK1.5, Biolegend, Cat.#: 100426), PerCP/Cy5.5 anti-CD4 (1:200, clone GK1.5, Biolegend, Cat.#: 100433), BV421 anti-CD8a (1:200, clone 53-6.7, Biolegend, Cat.#: 100737), FITC anti-CD8a (1:200, clone 53-6.7, Biolegend, Cat.#: 100706), PE-Dazzle594 anti-CD8a (1:200, clone 53-6.7, Biolegend, Cat.#: 100761), PE-Cy7 anti-CD8a (1:200, clone 53-6.7, Biolegend, Cat.#: 100721), BV605 anti-CD11b (1:200, clone M1/70, Biolegend, Cat.#: 101237), PE-Cy7 anti-CD11c (1:200, clone N418, Biolegend, Cat.#: 117317), BV570 anti-mouse/human-CD44 (1:100, clone IM7, Biolegend, Cat.#: 103037), BV711 anti-mouse/human-CD44 (1:100, clone IM7, Biolegend, Cat.#: 103057), FITC anti- mouse/human-CD44 (1:100, clone IM7, Biolegend, Cat.#: 103022), PerCP-Cy5.5 anti-mouse/human-CD44 (1:100, clone IM7, Biolegend, Cat.#: 103022, AF647 anti-CD45.1 (1:100, clone A20, Biolegend, Cat.#: 110720), PE anti-CD45.1 (1:100, clone A20, Biolegend, Cat.#: 110707), PE-Dazzle594 anti-CD45.1 (1:100, clone A20, Biolegend, Cat.#: 110747), PerCP/Cy5.5 anti-CD45.1 (1:100, clone A20, Biolegend, Cat.#: 110727), BV510 anti-CD45.2 (1:100, clone 104, Biolegend, Cat.#: 109837), FITC anti-CD45.2 (1:100, clone 104, Biolegend, Cat.#: 109805), PerCP-Cy5.5 anti-CD45.2 (1:100, clone 104, Biolegend, Cat.#: 109827), FITC anti-CD62L (1:100, clone MEL-14, Biolegend, Cat.#: 104405), PE-Dazzle594 anti-CD62L (1:100, clone MEL-14, Biolegend, Cat.#: 104447), FITC anti-CD103 (1:100, clone M290, BD Biosciences, Cat.#: 557494), APC anti-CD132/IL2RGc (1:100, clone TUGm2, Biolegend, Cat.#: 132307), PE-Dazzle594 anti-CD186/CXCR6 (1:200, clone SA051D1, Biolegend, Cat.#: 151116), PE anti-CX3CR1 (1:100, clone SA011F11, Biolegend, Cat.#: 149006), BV605 anti-CD279/PD-1 (1:100, clone 29F.1A12, Biolegend, Cat.#: 135219), BV421 anti-CD366/TIM-3 (1:200, clone RMT3-23, Biolegend, Cat.#: 119723), PerCP/Cy5.5 anti-TCRb (1:100, clone H57-597, Biolegend, Cat.#: 109227), AF700 anti-I-A/I-E (MHC class II) (1:500, clone M5/114.15.2, Biolegend, Cat.#: 107621), PE anti-H-2Kb bound to SIINFEKL (1:100, clone 25-D1.16, Biolegend, Cat.#: 141603), APC anti-human Granzyme B (1:200, clone GB12, Thermo Fisher Scientific, Cat.#: MHGB05), FITC anti-Ki-67 (1:100, clone SolA-15, Thermo Fisher Scientific, Cat.#: 11-5698-82), AF700 anti-Ki-67 (1:100, clone SolA-15, Thermo Fisher Scientific, Cat.#: 56-5698-82), PE anti-TCF1/TCF7 (1:40, clone S33-966, BD Biosciences, Cat.#: 564217), AF488 anti-pSTAT5 (0.03µg per test, clone 47/Stat5(pY694), BD Biosciences, Cat.#: 612598), eF660 anti-TOX (1:100, clone TXRX10, Thermo Fisher Scientific, Cat.#: 50-6502-82), eFluor660 Rat-IgG2a-k isotype-control (1:100, clone eBR2a, Thermo Fisher Scientific, Cat.#: 50-6502-82), APC Mouse-IgG1k isotype-control (1:200, clone P3.6.2.8.1, Thermo Fisher Scientific, Cat.#: 17-4714-42), AF488 Mouse-IgG1k isotype-control (0.03µg per test, clone MOPC-21, Biolegend, Cat.#: 400132), and rabbit-anti-mouse-TCF1/TCF7 (1:100, clone C.725.7, Thermo Fisher Scientific, Cat.#: MA5-14965) followed by AF647 Donkey-anti-rabbit IgG (1:200, clone Poly4064, Biolegend, Cat.#: 406414) or DL488 Donkey-anti-rabbit IgG (1:200, clone Poly4064, Biolegend, Cat.#: 406416).

The following antibodies were used for cell hashing for scRNA-seq and scTCR-seq analyses:
BRAFV600E into Ptger2-/-Ptger4fl/fl: dilution: 1:250, TotalSeq-C 0302, clone: M1/42;30-F11, Cat.#: 155863, Biolegend
BRAFV600E into CD4CrePtger2-/-Ptger4fl/fl : 1:250, TotalSeq-C 0303, clone: M1/42;30-F11, Cat.#: 155865, Biolegend
BRAFV600E into GzmBCrePtger2-/-Ptger4fl/fl: 1:250, TotalSeq-C 0304, clone: M1/42;30-F11, Cat.#: 155867, Biolegend
BRAFV600E into WT: 1:250, TotalSeq-C 0301, clone: M1/42;30-F11, Cat.#: 155861, Biolegend

The following antibodies were used for in vivo depletion of T cells in mice:
Anti-mouse CD4 (100 µg/mouse, clone GK1.5, BioXCell, Cat.#: BP0003-1)
Anti-mouse CD8ß (100 µg/mouse, clone 53-5.8, BioXCell, Cat.#: BE0223)

The following antibodies were used for in vivo blockade of IL-2 signalling in T cells:
Anti-mouse CD122 (300µg/mouse, clone TM-Beta 1, BioXCell, Cat.#: BE0298)
Anti-mouse CD132 (300µg/mouse, clone 3E12, BioXCell, Cat.#: BE0271)

If not stated otherwise, all antibodies were anti-mouse antibodies.

Validation

All antibodies listed in the previous section were validated by the manufacturer and/or by previous studies, and all primary antibodies were anti-mouse or anti-human antibodies. Mouse Cross-reactivity for anti-human antibodies was validated in-house using activation/stimulation assays in vitro with either mouse splenocytes or isolated CD8+ T cells from spleens.

Information on the validation of antibodies for flow cytometry can be found as stated below:

Biolegend antibodies: https://www.biolegend.com/en-us/quality/quality-control
Biolegend employs a comprehensive approach to antibody validation, analyzing 1-3 target cell types with single- and multi-colour analysis to encompass positive and negative cell types. Upon confirming specificity, each new lot is required to match the intensity of the in-date reference lot, with the brightness (MFI) evaluated across both positive and negative populations to ensure consistency. Furthermore, quality control testing, including a series of titration dilutions, is conducted for every lot.

Thermo Fisher Scientific antibodies: https://www.thermofisher.com/de/de/home/life-science/antibodies/invitrogen-antibody-validation.html
Thermo Fisher Scientific tests each antibody using different methods, including flow cytometry, Immunoprecipitation-Mass Spectrometry Antibody Validation, Knockout and Knockdown Antibody Validation, Independent Antibody Validation, Peptide Array Antibody Validation, Cell Treatment, Neutralization Antibody Validation, Relative Expression Antibody Validation, and SNAP-ChIP Antibody Validation. The precise validation method for each antibody is outlined in its respective antibody datasheet.

BD Biosciences antibodies: https://www.bdbiosciences.com/en-eu/products/reagents/flow-cytometry-reagents/research-reagents/quality-and-reproducibility
BD Biosciences tests each antibody on primary cells, cell lines or transfectant models using different methods, including flow cytometry, immunofluorescence, immunohistochemistry, or western blot. The precise validation method for each antibody is outlined in its respective antibody datasheet.

Validation and quality control of the TotalSeq-C 0301, TotalSeq-C 0302, TotalSeq-C 0303 and TotalSeq-C 0304 antibodies was carried out by Biolegend (https://www.biolegend.com/en-us/quality/quality-control) using flow cytometry (Cell-Surface Staining with Antibody: https://www.biolegend.com/protocols/cell-surface-flow-cytometry-staining-protocol/4283/) and sequencing as well as PCR to confirm the oligonucleotide barcodes (https://www.biolegend.com/en-us/quality/quality-control). Moreover, antibodies were validated in-house by flow cytometry using the PE anti-CD45 antibody (clone 30-F11, Biolegend, Cat.#: 103105) provided by Biolegend.

The anti-mouse CD4+ T cell-depleting antibody was validated by the manufacturer using western blot (https://bioxcell.com) and succesful depletion was validated in-house by flow cytometric staining of CD4+ T cells.
The anti-mouse CD8+ T cell-depleting antibody was confirmed to deplete CD8+ T cells by Inge Verbrugge et al., Cancer Research, 2012 and additionally validated in-house by flow cytotmetric analysis of CD8+ T cells.
The anti-mouse CD122 blocking antibody was validated by Sultan, H., et al., Cancer Immunol Res., 2019 for successful in vivo blocking of CD122 signalling.
The anti-mouse CD132 blocking antibody was validated by the manufacturer using western blot (https://bioxcell.com) and successful in vivo blocking of CD132 signalling was validated by Y. W. He et al., PNAS, 1995.

Eukaryotic cell lines

Policy information about cell lines and Sex and Gender in Research

Cell line source(s)

Control BRAFV600E, Ptgs1/Ptgs2-/- BRAFV600E cell lines were sourced from the Immunobiology Laboratory at The Francis Crick Institute, London, UK. Their generation has been previously described (Zelenay et al., Cell 2015).
The cell lines BRAFV600E-OVA and Ptgs1/Ptgs2-/-BRAFV600E-OVA were generated by lentiviral transduction as described in the methods section).
D4M.3A-pOVA cells were generated as previously described (Di Pilato et al., Nature 2019).
MC38-OVA and Panc02 cells (commercially available from Cytion) were provided by Veit R. Buchholz (Institute of Medical Microbiology, Immunology, and Hygiene, Technische Universität München (TUM).
MC38 (commercially available from Cytion) were provided by Achim Krüger (Institute of Experimental Oncology, TUM).

Authentication

For Ptgs1/Ptgs2-/- BRAFV600E cells, absence of PGE2 production was routinely confirmed by PGE2 ELISA (Cayman).
There were no further authentications conducted in the laboratory.

Mycoplasma contamination

All cell lines were routinely tested negative for mycoplasma contamination in-house by PCR.

Commonly misidentified lines
(See ICLAC register)

No commonly misidentified cell lines were used in this study.

Animals and other research organisms

Policy information about studies involving animals; ARRIVE guidelines recommended for reporting animal research, and Sex and Gender in Research

Laboratory animals	The following strains of mus musculus were used:<br>Wildtype mice: C57BL/6J (strain #000664)<br>OT-I mice: C57BL/6-Tg(TcraTcrb)1100Mjb/J (strain #003831)<br>CD45.1 mice: B6.SJL-Ptprca Pepcb/BoyJ (strain #002014)<br>Ptger2−/− mice: B6.129-Ptger2tm1Brey/J (strain #004376)<br>Rag1−/− mice: B6.129S7-Rag1tm1Mom/J (strain #002216)<br>Ptger4fl/fl mice: B6.129S6(D2)-Ptger4tm1.1Matb/BreyJ (strain #028102)<br>CD4Cre mice: B6.Cg-Tg(Cd4-cre)1Cwi/BfluJ (strain #022071)<br>GzmBCre mice: B6;FVB-Tg(GZMB-cre)1Jcb/J (strain #003734)<br><br>If not stated otherwise, all mice were used on a CD45.2/CD45.2 background. All mice were maintained and bred at the Klinikum rechts der Isar, Technical University of Munich, TUM or at the at Klinikum der Universität München, Ludwig-Maximilians-University, (LMU) under specific-pathogen-free conditions. The following mouse strains were additionally bred by Charles River (Calco, Italy): Ptger2-/-Ptger4fl/fl, CD4CrePtger2-/-Ptger4fl/fl. Mice were housed under controlled conditions, including a 12-hour light-dark cycle, with an ambient temperature of 24°C, and humidity maintained at 55%. Mice were used at 6-12 weeks of age.
Wild animals	No wild animals were used in this study.
Reporting on sex	In all experiments, mice of the same age were sex-matched and randomly assigned to experimental and control groups.<br>The results in this study are not restricted to one sex.
Field-collected samples	No field-collected samples were used in this study.
Ethics oversight	All mice were kept according to the guidelines of the Federation of European Laboratory Animal Science Associations and experiments were authorised by permission of the Government of upper Bavaria (Department 5).

Note that full information on the approval of the study protocol must also be provided in the manuscript.

Flow Cytometry

Plots

Confirm that:

☒ The axis labels state the marker and fluorochrome used (e.g. CD4-FITC).

☒ The axis scales are clearly visible. Include numbers along axes only for bottom left plot of group (a 'group' is an analysis of identical markers).

☒ All plots are contour plots with outliers or pseudocolor plots.

☒ A numerical value for number of cells or percentage (with statistics) is provided.

Methodology

Sample preparation	Cell isolation for flow cytometry and cell sorting:<br>Tumour or organ weight was determined using a microscale. Tumour samples were mechanically dissociated and incubated with Collagenase IV (200 U/ml) and DNase I (100 µg/ml) for 40 min at 37 °C and filtered through a 70 µm and a 30 µm cell strainer to generate single-cell suspensions. Spleens were passed through a 70 µm cell strainer, followed by red blood cell lysis and a second filtration step using a 30 µm cell strainer. Lymph nodes were passed through a 30 µm cell strainer. For the isolation of migratory cDC1, lymph nodes were processed as described for tumour samples.<br><br>For in vitro generation of repetitively activated TCF1+CD8+ T cells, naive splenic CD8+ T cells from WT mice were stimulated with mouse anti-CD3/CD28 microbeads and low dose IL-2 (85 U/ml) for two consecutive 48 h cycles. Cells were purified by gradient centrifugation (Pancoll) and used in experiments as indicated before staining with fluorescently-labelled antibodies in FACS buffer and flow cytometric analysis.<br><br>For further details, please see the methods section of the manuscript.
Instrument	Cells were analysed on a LSRFortessa Cell Analyzer (BD Biosciences) a SP6800 Spectral Cell Analyzer (SONY Biotechnologies) or a SA3800 Spectral Cell Analyzer (SONY Biotechnologies).<br><br>Naive CD8+ T cells were sorted using the SH800 Cell Sorter (SONY Biotechnology).<br>Tumor infiltrating CD8+ T cells were sorted using BD FACSAria III Cell Sorter (BD Biosciences).<br><br>Reverse transcription of RNA into cDNA was performed using a ProFlex PCR System (Thermo Fisher Scientific).<br>Quantitative real-time PCR was carried out on a LightCycler 480 (Roche).

Software	The following softwares were used: SP6800 Sepctral Analyzer Software v2.0.2.14140 (SONY Biotechnology), SA3800 Spectral Analyzer Software v2.0.5.54250 (SONY Biotechnology), SH800 Cell Sorter Software v2.1.6 (SONY Biotechnology), BD FACSDiva™ Software v9.0.1 and v8.0.1 (BD Biosciences), LightCycler 480 SW v1.5.1 (Roche).<br><br>All obtained data from flow cytometry was analysed with the FlowJo software (BD Biosciences, v10.8.1 and v10.8.2).
Cell population abundance	For the adoptive T cell transfers and scRNA sequencing + scTCR sequencing, respective cell populations were sorted using the "high purity" mode. Sorted fractions were assessed for purity and viability by flow cytometry. Purity of sorted naive and tumor-infiltrating CD8+ T cells was >95%.
Gating strategy	After gating on live, single cells, immune cell populations were defined the following:<br><br>CD8+ T cells: CD45+CD8+TCRb+ or CD45+CD8+CD3+. Congenically marked cells were identified based on CD45.1/2 staining as indicated in the figure legends. T cell subpopulations were identified using the following additional markers: CD44, TIM-3, CXCR6, TCF1, CD62L, TOX, Ki-67, and GzmB<br><br>CD4+ T cells: CD45+CD4+TCRb+<br><br>Migratory cDC1 in tdLN: CD45+CD11c+MHCIIhiCD103+CD8a–CD11b–<br><br>In vitro generated repetitively stimulated TCF1+CD8+ T cells: CD45+CD8+TCRb+ or CD45+CD8+CD3+<br>Naive OT-I T cells from blood for adoptive T cell transfers: CD45.1+CD8a+CD62L+CD44low

☒ Tick this box to confirm that a figure exemplifying the gating strategy is provided in the Supplementary Information.
